# Expression and Trans-Specific Polymorphism of Self-Incompatibility RNases in *Coffea* (Rubiaceae)

**DOI:** 10.1371/journal.pone.0021019

**Published:** 2011-06-22

**Authors:** Michael D. Nowak, Aaron P. Davis, François Anthony, Anne D. Yoder

**Affiliations:** 1 Department of Biology, Duke University, Durham, North Carolina, United States of America; 2 Royal Botanic Gardens, Kew, Richmond, Surrey, United Kingdom; 3 Institut de Recherche pour le Développement, centre de Montpellier, Unité Mixte de Recherche “Résistance des Plantes aux Bioagresseurs”, Montpellier, France; The University of Queensland, St. Lucia, Australia

## Abstract

Self-incompatibility (SI) is widespread in the angiosperms, but identifying the biochemical components of SI mechanisms has proven to be difficult in most lineages. *Coffea* (coffee; Rubiaceae) is a genus of old-world tropical understory trees in which the vast majority of diploid species utilize a mechanism of gametophytic self-incompatibility (GSI). The S-RNase GSI system was one of the first SI mechanisms to be biochemically characterized, and likely represents the ancestral Eudicot condition as evidenced by its functional characterization in both asterid (Solanaceae, Plantaginaceae) and rosid (Rosaceae) lineages. The S-RNase GSI mechanism employs the activity of class III RNase T2 proteins to terminate the growth of “self” pollen tubes. Here, we investigate the mechanism of *Coffea* GSI and specifically examine the potential for homology to S-RNase GSI by sequencing class III RNase T2 genes in populations of 14 African and Madagascan *Coffea* species and the closely related self-compatible species *Psilanthus ebracteolatus*. Phylogenetic analyses of these sequences aligned to a diverse sample of plant RNase T2 genes show that the *Coffea* genome contains at least three class III RNase T2 genes. Patterns of tissue-specific gene expression identify one of these RNase T2 genes as the putative *Coffea* S-RNase gene. We show that populations of SI *Coffea* are remarkably polymorphic for putative S-RNase alleles, and exhibit a persistent pattern of trans-specific polymorphism characteristic of all S-RNase genes previously isolated from GSI Eudicot lineages. We thus conclude that *Coffea* GSI is most likely homologous to the classic Eudicot S-RNase system, which was retained since the divergence of the Rubiaceae lineage from an ancient SI Eudicot ancestor, nearly 90 million years ago.

## Introduction

Many bisexual flowering plants avoid the deleterious effects of inbreeding by employing genetically controlled self-incompatibility (SI) mechanisms to ensure outcrossing [1]. SI mechanisms provide the biochemical machinery necessary for plants to recognize and reject their own pollen as well as non-self pollen with a genotype sufficiently similar to elicit activation of the SI mechanism. SI plants thus require a pollen donor with a divergent genotype for successful fertilization, a mating system known as “obligate outcrossing” [2]. SI plays an important role in shaping the spatial and temporal distribution of genetic diversity in plant populations and is thought to influence patterns of lineage diversification in clades within which these mechanisms are utilized [3], [4].

It is likely that more than half of all flowering plant species employ biochemical SI mechanisms [5], [6], but identifying the loci involved in SI (S-loci) has proven to be difficult [7]. In flowering plants, the biochemical components of only three SI mechanisms have been well-characterized (see [8] for review): 1) the Papaveraceae calcium signaling system [9]–[11]; 2) the Brassicaceae sporophytic SRK and SP11/SCR SI system [12], [13]; and 3) the Eudicot gametophytic S-RNase system [14]. The unique biochemical mechanisms characterized in both the Papaveraceae and Brassicaceae point to independent evolution of these SI systems in the respective ancestors of these families. In contrast, the S-RNase system is known to function in both of the two major Eudicot clades: the asterids (i.e. Solanaceae and Plantaginaceae), and the rosids (i.e. Rosaceae). Furthermore, there is now reliable phylogenetic evidence for homology of the S-RNase gene between these three families that strongly supports a single evolutionary origin for the S-RNase SI mechanism in an ancient Eudicot lineage ancestral to both asterids and rosids [15]–[17]. The S-RNase SI mechanism is a type of gametophytic self-incompatibility (GSI) that functions through the selective de-activation of RNase cytotoxicity in non-self pollen tubes as they grow through the style. The S-locus in Eudicot species utilizing the S-RNase GSI system carries genes with reproductive tissue-specific expression patterns (i.e. pistil or pollen tube; [18]–[22]). The S-locus gene expressed in the pistil (i.e. the female floral organ) was the first component of the S-locus to be identified [23], and was found to encode a ribonuclease in the RNase T2 protein family [24]. While species in at least 23 other Eudicot families are known to employ some mechanism of GSI [3], there has been a conspicuous lack of published data from any of these families that would demonstrate utilization of the ancestral Eudicot S-RNase SI mechanism.

We present here the first molecular evidence for the utilization of the ancestral Eudicot S-RNase system in the asterid genus *Coffea* L. (Rubiaceae). *Coffea* is a genus of substantial agricultural and commercial importance [25], with two species, *C. arabica* L. (Arabica) and *C. canephora* A. Froehner (Robusta), used in the production of the beverage coffee. There are approximately 103 species in the genus, most of which are understory trees, distributed in the tropical belt and found in Africa, Madagascar, the Mascarene Islands, and the Comoros Islands [26], [27]. The vast majority of *Coffea* species are known to exhibit a strong GSI response, but three African species (*C. arabica*, *C. anthonyi* Stoff. & F.Anthony, and *C. heterocalyx* Stoff.) are exceptional for their ability to self-fertilize (i.e. self-compatibility, SC) [26], [28], [29]. Parenthetically, the genus *Psilanthus*, a genus of ca. 22 species that is very closely allied to *Coffea* [30], is known to contain at least one SC species, viz. *P. ebracteolatus* Hiern [31]. *C. arabica* is the allotetraploid product of recent hybridization between the two African species *C. canephora* (west and central Africa) and *C. eugenoides* (central Africa) [30], [32], [33]. Self-compatibility in *C. arabica* is thus not surprising given the strong correlation between polyploidy and the breakdown of SI previously documented in other angiosperm lineages (e.g. see [34]–[37]). In contrast to the unique polyploid origin of *C. arabica*, the cause of SC in diploid *C. heterocalyx* and *C. anthonyi* is unknown. Coulibaly et al. [38] exploited *C. heterocalyx* SC to genetically map loci implicated in the *C. canephora* GSI mechanism. A single 5.4 cM *Coffea* self-incompatibility locus (S-locus) was identified, but they were unable to fine map or sequence this locus to identify individual genes.

We present evidence from several sources of data that the *Coffea* GSI mechanism is homologous to the S-RNase GSI system employed by many Eudicot lineages. To assess the homology of the *Coffea* GSI mechanism to S-RNase GSI, we collected sequence polymorphism data from RNase T2 genes expressed in floral tissues from SI and SC *Coffea* species. We compare three specific characteristics of these data to that of previously isolated S-RNase genes. 1) Phylogenetic analyses consistently place all S-RNase genes within the Class III RNase T2 gene family. 2) Populations of SI plants harbor exceptional allelic diversity and polymorphism at the S-RNase locus. Phylogenetic analyses of S-RNase alleles isolated from related species show that allelic lineages are often shared between species, a pattern known as trans-specific polymorphism. Such phylogenetic patterns are thought to represent the maintenance of ancestral S-allelic diversity through strong negative frequency-dependant selection [39]. 3) Distinct from other Class III T2 RNases, expression of S-RNase genes is generally limited to floral tissues. Below, we describe in detail these characteristics of the S-RNase GSI system and the means by which they are employed to test for homology to the *Coffea* GSI mechanism.

### Evidence for S-RNase GSI homology

#### S-RNase genes are members of the class III RNase T2 gene family

Plant RNase T2 proteins are endoribonucleases with selective activity expressed in a diverse array of tissues in response to physical or pathogen-induced stress [40], [41]. RNase T2 proteins have been the subject of many detailed biochemical and functional studies [40], [42], and contain several conserved active sites (CAS), which are amino acid motifs involved in the biochemistry of ligand-specific RNase activation [43]. Plant RNase T2 genes are grouped into three classes (i.e. class I, II, and III) based on conserved structural motifs and evolutionary history as defined by phylogenetic placement. Class I and II RNase T2 genes are either expressed in response to cellular stress (Class I) or constitutively expressed (Class II). Class III RNase T2 genes contain either one or two introns, and carry conserved amino acid motifs that have been shown to involved in activating RNase function [17], [40], [41]. All S-RNase genes known to function in the S-RNase GSI system encode proteins in the Class III RNase T2 subfamily. S-RNase expression is generally limited to floral tissues [44], [45], but Class III RNases that are unrelated to the GSI mechanism (i.e. those isolated from sequenced genomes and EST libraries of SC Eudicot species) tend to exhibit little tissue-specificity in expression and their cellular and biochemical function is generally unknown [41]. These genes have been termed “S-like” [17] or “proto-S-RNases” [41] due to domain structure similarity and phylogenetic placement within the Class III RNase T2 family. We employ phylogenetic analysis to test for the placement of putative *Coffea* S-RNase genes within the Class III RNase T2 family.

#### S-RNase genes exhibit deep allelic divergence and trans-specific polymorphism

**Figure 1:**
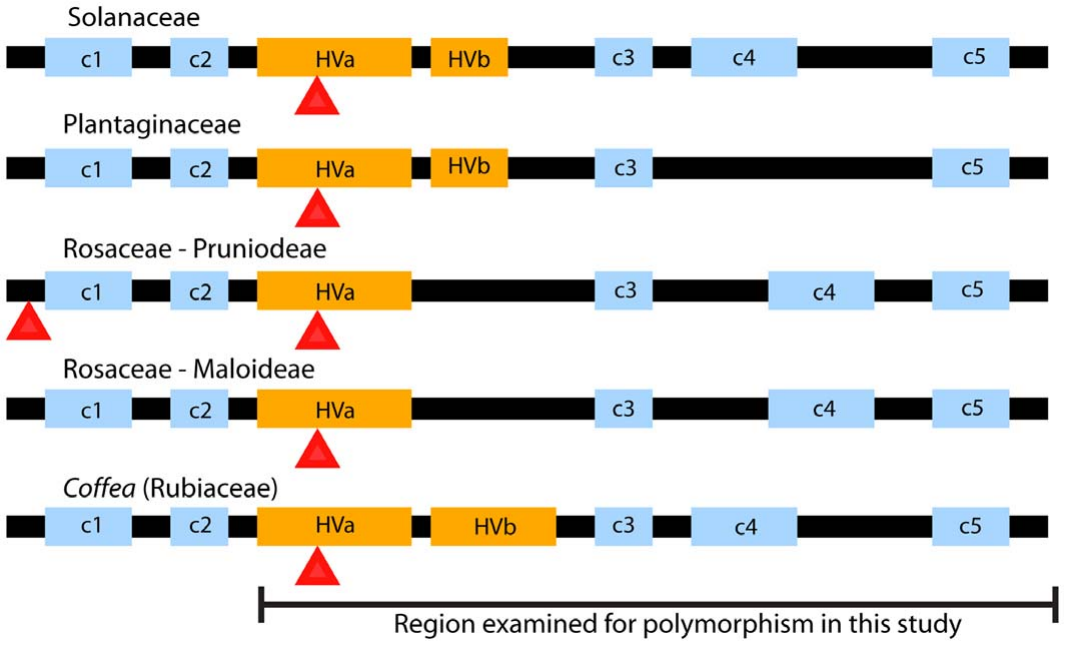
Eudicot S-RNase protein conserved domain structure. The conserved domain structures of S-RNases employed in GSI Eudicot lineages and the conserved domains revealed in this study of *Coffea* are labeled accordingly. Canonical S-RNase conserved regions are labeled “c1”–“c5”, and hyper-variable regions are labeled “HVa” and “HVb”. The red arrowheads below the protein constructs indicate intron positions. Intron position data and presence of conserved region c1 was validated in *Coffea* based on genomic sequence and 5′RACE data from *C. arabica* and *C. canephora* alone (data not shown). Results relating to RNase polymorphism reported in this study are based on 3′RACE data from the hyper-variable regions (i.e. HVa) to the end of transcripts (i.e. through c5). This figure was adapted from Vieira and Charlesworth [91].

S-RNase genes contain five conserved regions distinguished by specific amino acid motifs (denoted c1 to c5), and between c2 and c3 carry either one (Solanaceae and Plantaginaceae) or two (Rosaceae) regions of elevated sequence variation, or “hypervariable” regions (see Figure 1). Sites within the hypevariable regions appear to be experiencing positive selection (i.e. sites with dN/dS ratios >1.0), suggesting a possible implication in the biochemical trigger of self-recognition [39], [46], [47], [48], [49]. Beyond the extremely divergent hypervariable regions, S-RNase alleles display characteristically deep sequence divergence and exceptional levels of polymorphism in SI populations [50]. If *Coffea* GSI is homologous to the S-RNase GSI system, putative S-RNase alleles isolated from *Coffea* populations should show a level of sequence divergence and polymorphism consistent with known S-RNase alleles. Additionally, theoretical expectations and empirical evidence suggest that diploid SC *Coffea* species should show dramatically reduced S-allele polymorphism as the loss of SI will be accompanied by an extreme genetic bottleneck at the S-locus [51], [52].

The S-RNase GSI mechanism places the S-locus under negative frequency-dependent selection [39], [53], [54], which means that the frequency of an S-allele is inversely related to that allele's effect on fitness. Individuals in an SI population carrying rare S-allele genotypes benefit from an abundant supply of potential mates (i.e. pollen donors or recipients) and thus experience a significant fitness advantage. The result of this negative frequency-dependant selection is that the fecundity of an individual within an SI population is inversely proportional to the frequency of that individual's S-alleles in the population. These conditions have important ramifications for the frequency distribution of S-alleles in finite SI populations: the probability that an allele will be fixed is zero, and while S-alleles will eventually be replaced by new specificities, the time-scale of this replacement is far greater than at neutral loci [39], [55], [56]. Thus, ancestral polymorphisms at the S-locus will be maintained in the population much longer than at neutral loci. As a result, phylogenetic analyses of S-alleles show a pattern of trans-specific polymorphism [39], [50], [57], [58], similar to empirical studies of genes thought to be evolving under negative frequency-dependant selection in animals (e.g. primate MHC alleles; [59]), and fungi (e.g. mating pheromone receptors; [60]). If *Coffea* GSI is homologous to the Eudicot S-RNase GSI mechanism, putative S-RNase alleles from *Coffea* species should show a clear pattern of trans-specific polymorphism reflecting ancestral allelic diversity.

#### S-RNase expression is limited to pistil tissues

The existence of S-like class III RNase T2 genes in SC Eudicot lineages suggests that a more rigorous assay of homology is required beyond phylogenetic placement alone. While S-RNase genes are expressed primarily in female floral organs (i.e. the pistil; [44], [45]), S-like RNase genes generally show little tissue-specific expression (i.e. they are expressed constitutively; [41]). Therefore, if we have correctly identified the *Coffea* S-RNase, we expect to find expression limited to pistil tissues, whereas putative S-like RNase T2 genes are unlikely to display the same tissue-specificity.

## Results

### African and Madagascan *Coffea* species exhibit strong gametophytic self-incompatibility

**Figure 2:**
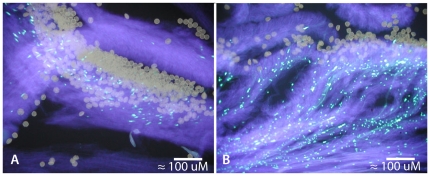
*Coffea* gametophytic self-incompatibility. The strength of the *Coffea* GSI response was measured by examining pollen tube growth using analine blue staining. A. Self-pollen tube growth in SI *C. perrieri* in cultivation at the Kianjavato Coffee Research Station, Madagascar. Pollen tubes terminate shortly after entering the style in bulbous callose plugs that fluoresce brightly. B. Self-pollen tube growth in SC *C. arabica* in cultivation at the Duke University Department of Biology Greenhouse Facility. Pollen tubes can be seen growing through the style tissue unhindered, leading to self-fertilization.

Microscopic observations of self pollen tube growth in *C. canephora* were found to be consistent with a gametophytic SI mechanism [38], [61], but SI in all other *Coffea* species has been assumed by the observation of reduced seed set in self versus outcross pollinations [62], [63]. While this is a valid metric for evaluating the strength of SI, inbreeding depression can also lead to significant reductions in seed set [1], [64], [65] which could confound the conclusions of these previous mating system experiments in *Coffea*. Therefore, to examine the strength of the GSI response in several species of *Coffea* (Table 1), we performed self-fertilizations and observed pollen tube growth microscopically with a standard aniline blue staining procedure [66]. Example micrographs of pollen tube growth resulting from self-compatible and incompatible fertilizations are shown in Figure 2. We were able to microscopically examine the GSI response in about 70% of the individual *Coffea* trees utilized in this study, including at least one representative of each species. All putatively SI *Coffea* species examined show a characteristic termination of self pollen tube growth with a bulbous mass of callose tissue after growing a few millimeters into the style tissue (Figure 2A)[67]. These results are consistent with a strong gametophytic self-incompatibility response as previously documented both in *C. canephora* [38] and other GSI species [9]. Figure 2B shows an example of self pollen tubes that are unhindered in their passage through the style, which was observed in the SC species *C. arabica, C. heterocalyx,* and *Psilanthus ebracteolatus*. These results are consistent with all previous reports of self-compatibility in these species [26], [29].

**Table 1:** S-RNase alleles isolated from *Coffea* and *Psilanthus*.

Species	Distribution	SI/SC	Plants Sampled	S-RNase Alleles
*C. andrambovatensis*	E. Madagascar	SI	4	S1, S4, S8, S21, S36, S37
*C. bonnieri*	N. Madagascar	SI	3	S13, S25, S26, S27, S45
*C. millotii*	E. Madagascar	SI	8	S8, S10, S17, S18, S19, S32, S33
*C. montis-sacri*	E. Madagascar	SI	4	S9, S13, S18, S21, S24, S34
*C. perrieri*	W. Madagascar	SI	11	S11, S12, S13, S15, S16, S26
*C. resinosa*	E. Madagascar	SI	7	S1, S4, S10, S13, S25, S28, S29, S51
*C. tricalysoides*	N. Madagascar	SI	2	S11, S38, S39
*C. tsirinananae*	N. Madagascar	SI	5	S6, S7, S8, S9, S10, S49
*C. brevipes*	Tropical W. Africa	SI	1	S1
*C. canephora*	Tropical W. and central Africa	SI	3	S3, S17, S27, S41, S42
*C. eugenoidoides*	Tropical central Africa	SI	4	S1, S4, S13
*C. pseudozanguebariae*	Tropical E. Africa	SI	1	S7, S38
*C. arabica*	Tropical NE Africa	SC*	1	S1, S2**, S3
*C. heterocalyx*	Tropical W. Africa	SC	4	S12
*P. ebracteolatus*	Tropical W. Africa	SC	1	PS1

*SC likely the result of allotetraploid origin (i.e. 2n = 44).

**Short message length indicates that this is likely pseudogene copy.

### The *Coffea* genome contains at least three Class III RNase T2 genes

**Figure 3:**
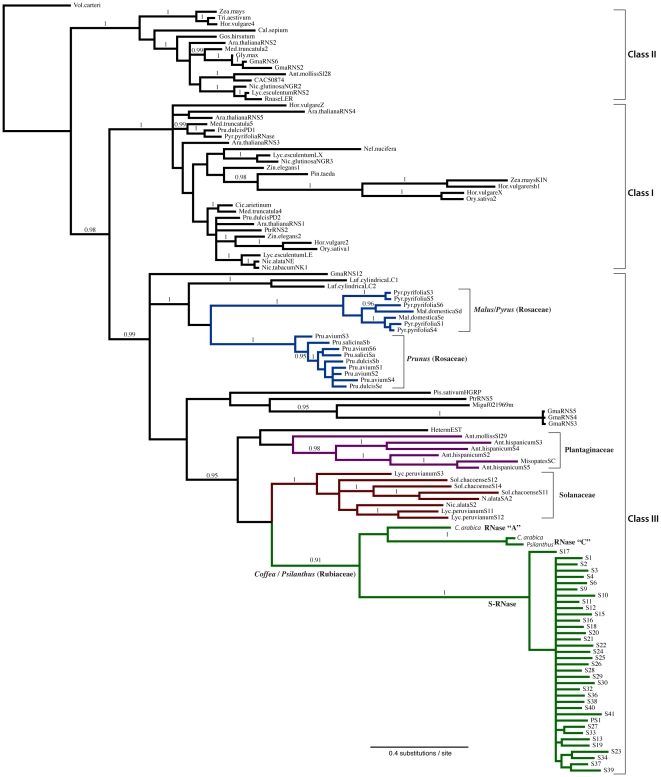
Phylogenetic relationships of plant RNase T2 genes. The maximum *a posteriori* tree generated from a Bayesian phylogenetic analysis of RNase T2 amino acid sequences. Branches receiving a posterior probability of 0.95 or greater have been labeled. The Class III RNase T2 genes form a well-supported clade sister to an unresolved clade of Class I and II RNase T2 genes. S-RNase alleles from other GSI lineages are labeled and color-coded (Solanaceae in red; Plantaginaceae in purple; Rosaceae in blue), and all Class III S-Like RNases as well as all Class I and II RNases are shown in black. RNase T2 genes isolated from *Coffea* and *Psilanthus* species are shown in green. The tree shows that the *Coffea* genome contains three distinct RNase T2 genes labeled “A”, “C” and S-RNase. Note the significant sequence divergence (i.e. represented by long terminal branch lengths) between putative alleles of the S-RNase gene.

In order to test if *Coffea* species utilize a GSI mechanism homologous to the canonical Eudicot S-RNase system, we cloned RNase T2 genes expressed in pistil tissues (i.e. the female floral organ) from 14 species of African and Madagascan *Coffea* and the related species *P. ebracteolatus*. Visual analysis of the sequences isolated from *C. arabica* revealed three distinct types of RNase T2 genes, which we refer to here as RNase “A”, RNase “C”, and S-RNase. We aligned all of the unique RNase T2 sequences (i.e. putative alleles) generated through our cloning strategy with a large sample of plant RNase T2 genes and performed a Bayesian phylogenetic analysis to examine the molecular evolution and class membership of RNase T2 genes in the *Coffea* genome. The resulting phylogenetic tree, shown in Figure 3, exhibits well-supported clades of Class I, II, and III T2 RNases. Furthermore, the placement of RNase T2 genes that we have isolated from the *Coffea* genome suggests that it contains at least three RNase T2 gene copies (i.e. RNase “A”, RNase “C”, and S-RNase), with unambiguous membership in the Class III RNase T2 subfamily as defined by Sassa et al. [24]. Additionally, RNase T2 cDNAs that we have isolated from *P. ebracteolatus* show clear homology to *Coffea* RNase “C” and S-RNase genes, but this species appears to lack a homolog to the *Coffea* RNase “A” gene.

All previously identified RNase genes implicated in the Eudicot S-RNase GSI system are members Class III RNase T2 family, and the proteins they encode are known to be relatively basic (i.e. isoelectric point, or pI>7.5), while Class I and II RNase T2 proteins tend to be relatively acidic (i.e. pI<7.5; [41]). To further evaluate the class III membership of *Coffea* RNase T2 genes, we calculated the predicted pI of all partial transcripts from conserved active site I (CAS I as defined by Irie [68]) to the end of the transcript. Consistent with class III RNase T2 membership, predicted proteins from *C. arabica* and *P. ebracteolatus* RNase “A”, “C” genes are distinctly basic (8.85 and 8.43, respectively) and all putative S-RNase transcripts are also basic (mean pI = 8.97, standard deviation = 0.38).

### *Coffea* S-RNase heterozygosity

Of the 60 individual *Coffea* trees sampled in our study, all but 10 specimens from GSI species (see above) were found to carry two putatively functional S-RNase alleles (i.e. assumed by the absence of premature stop codons or frame-shift mutations). It is likely that amplification efficiency will vary considerably when employing degenerate primers. The seeming homozygosity observed in 10 SI *Coffea* specimens is likely a product of over-representation of one of the two alleles actually carried by these individuals in the initial PCR, and thus simply a limitation of our cloning and amplification strategy rather than true S-RNase homozygosity. Supporting this claim, when we screened amplicon pools more deeply from one of these putatively homozygous individuals (i.e.>48 colonies sequenced), a second S-RNase allele was found. All four specimens of the SC species *C. heterocalyx* were found to be homozygous for allele S12 despite relatively deep screening of amplicon pools (i.e. approximately 48 clones were sequenced) from two individual plants. The SC species *P. ebracteolatus* was also found to be homozygous for an S-RNase allele (PS1). The single accession of the tetraploid SC species *C. arabica* sampled in our study was found to carry three putative S-RNase alleles (i.e. S1, S2, and S3), but one of these (i.e. S2) was a short transcript that may represent a pseudogene copy.

### Allelic divergence and patterns of polymorphism at the *Coffea* S-RNase

**Figure 4:**
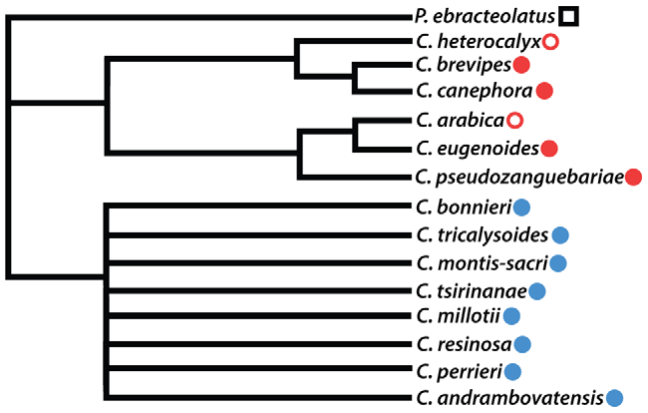
*Coffea* species tree. The tree is shown rooted with the outgroup representative *Psilanthus ebracteolatus*. The geographic origin of species is shown in the color-coded symbols (African species in red, Madagascan species in blue, *Psilanthus* in black). Species with filled symbols are self-incompatible (SI; e.g. *C. canephora*), and species with open symbols are self-compatible (SC; e.g. *C. arabica*). The phylogenetic relationships presented here are summarized from the results of previously published phylogenetic studies in the genus based on chloroplast and nuclear sequence data [30], [31].

**Figure 5:**
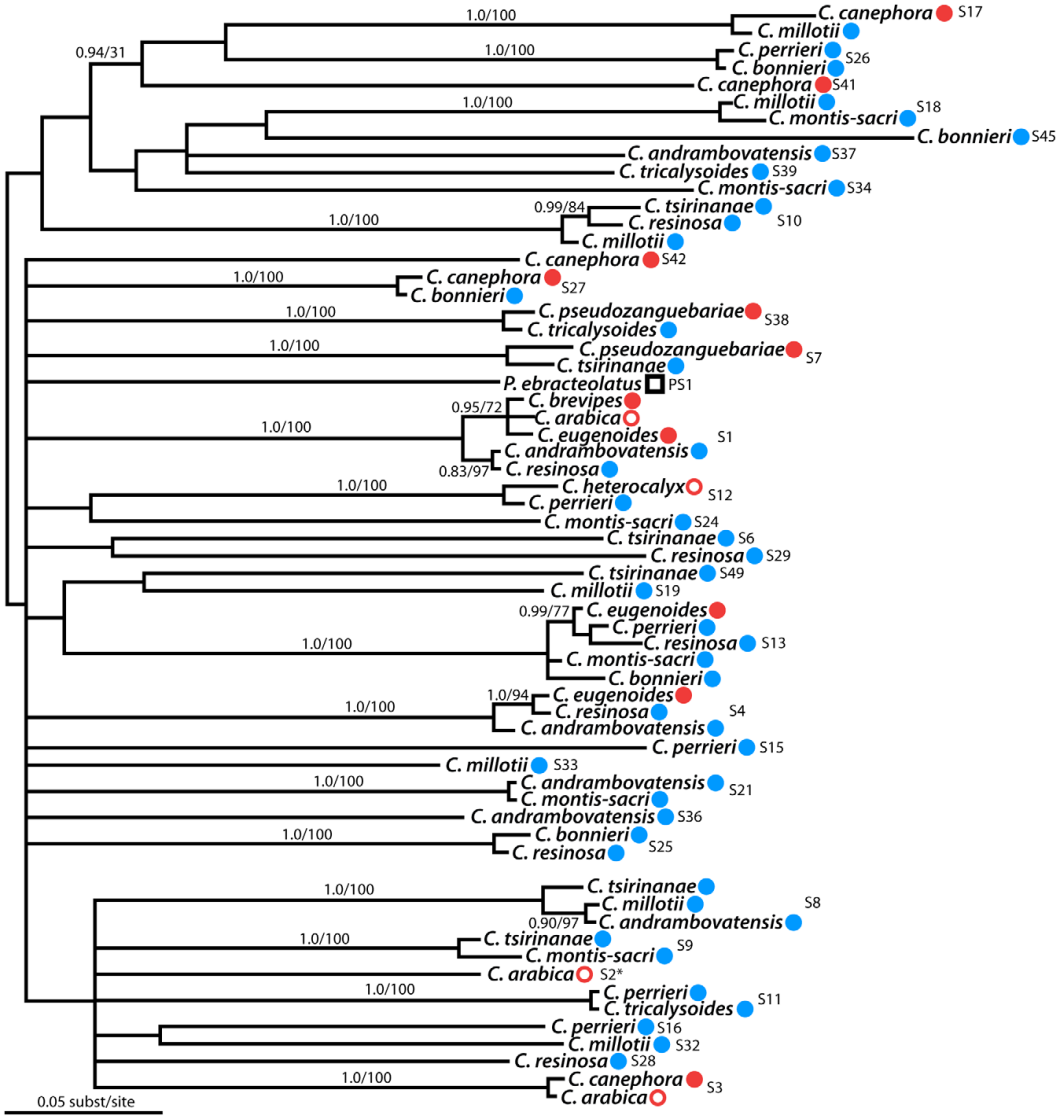
*Coffea* S-RNase trans-specific polymorphism. A. The maximum a posteriori phylogram of putative *Coffea* S-RNase alleles is shown with midpoint rooting and branches receiving strong support are labeled (i.e.>95% posterior probability). The *Psilanthus* S-RNase homolog is indicated by a black box. The geographic origin of putative S-RNase alleles is indicated by the colored symbols following the labeling scheme in Figure 4 (African = red; Madagascan = blue). All putative S-RNase allelic lineages (i.e. sequences that consistently group together and exhibit minimal divergence) have been labeled on the tree (e.g. S1, S4, etc.). All individuals from the self-compatible species *C. heterocalyx* carry sequences from just one putative S-RNase allelic lineage (S12). The gene tree shows a clear signature of trans-specific polymorphism in putative S-RNase alleles sequenced from a phylogenetically and geographically diverse sample of *Coffea* species.

Putative S-RNase sequences isolated from samples of *Coffea* and *Psilanthus* were sorted into 36 putative allelic lineages based on nearly identical nucleotide composition, while a mean pairwise genetic distance of 0.248 (standard deviation = 0.064) was found between putative S-RNase allelic lineages. Using the maximum likelihood method employed in the paml software package [69], pairwise dN/dS ratios between putative *Coffea* S-RNase allelic lineages are estimated to be between 0.15 and 0.87 (mean dN/dS = 0.36; see Table S4, Supporting Information Files). We constructed a gene tree of putative *Coffea* S-RNase alleles isolated from each species sampled. Visual comparison of this gene tree with the most likely species tree of the *Coffea* species in our study and *P. ebracteolatus* (Figure 4) provides a test for trans-specific polymorphism of the putative *Coffea* sequences. The S-RNase gene tree shown in Figure 5 deviates significantly from the branching pattern represented in the species tree, showing that many S-RNase allelic lineages exist in distantly related clades of *Coffea* (i.e. African and Madagascan species), which is a pattern indicative of trans-specific polymorphism. This pattern is perhaps most striking when considering S-RNase allelic lineages shared between species of African and Madagascan *Coffea*, which have been shown to represent deeply divergent clades in the genus [30], [31].

### Expression patterns exhibited by RNase T2 genes in *Coffea*

**Figure 6:**
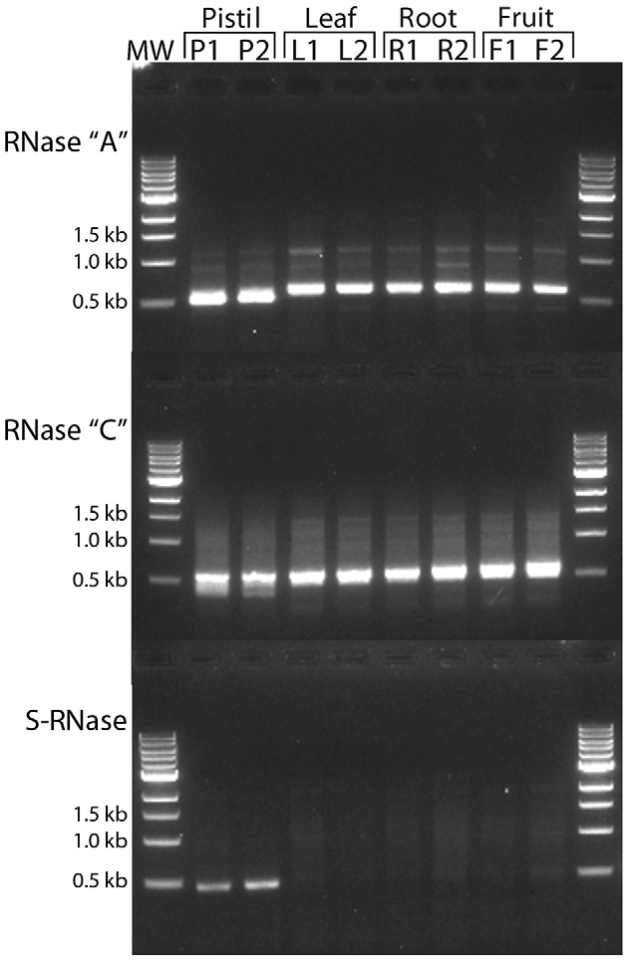
Expression of RNase T2 genes in *C. arabica* tissues. Agarose gel image showing RT-PCR results using gene-specific primers for *Coffea* RNase “A”, “C”, and S-RNase genes. cDNAs were generated from RNA extracts of various *C. arabica* tissues labeled as follows: P1 = pre-anthesis pistils dissected from flower buds; P2 = post-anthesis pistils; L1 = leaf at 1 week; L2 = leaf at >2 weeks; R1 = fine root; R2 = primary root; F1 = fruit at approximately 1 week; F2 = fruit at >3 weeks. RNase “A” and “C” genes are constitutively expressed in *C. arabica* tissues, but the S-RNase gene shows pistil-specific expression characteristic of genes involved in the S-RNase GSI system.

While the placement of the *Coffea* “A”, “C”, and S-RNase genes within the class III RNase T2 subfamily is consistent with the S-RNase GSI system, we would expect that an S-RNase would also show gene expression isolated to floral tissues, which is a characteristic of all known S-RNase genes. In order to determine which, if any, of the three class III RNase T2 genes expressed in *Coffea* pistils is most likely the S-RNase gene implicated in the GSI mechanism, we examined expression patterns of each gene in various tissues from a single *C. arabica* tree using standard RT-PCR procedures. Figure 6 shows that the *Coffea* “A” and “C” class III RNase T2 genes are constitutively expressed in relatively equal proportion in all tissues examined (i.e. pistils, leaves, roots, and fruits) regardless of developmental stage. In striking contrast to the constitutive expression patterns of the RNase “A” and “C” genes, Figure 6 shows clearly that expression of the putative *Coffea* S-RNase gene is limited to pistil tissues.

## Discussion

S-RNase genes that have been implicated in the S-RNase-based GSI systems of Solanaceae, Plantaginaceae, and Rosaceae are members of the class III RNase T2 gene subfamily. The results of our phylogenetic analysis of RNase T2 genes indicate the existence of at least three distinct class III RNase T2 genes (i.e. RNase “A”, “C”, and S-RNase) in the *Coffea* genome. We have also shown that the genome of the SC species *P. ebracteolatus* lacks a homolog to the *Coffea* RNase “A” gene. The phylogenetic relationships of the three class III RNase T2 genes supports a more recent ancestry of *Coffea* RNase “A” and “C” genes. Taken together, these data suggest that at some point after the divergence of *Coffea* from the ancestral lineage of *Coffea* and *Psilanthus* the RNase “C” gene was duplicated (i.e. producing the *Coffea* RNase “A” gene) and in the time since duplication, the two gene copies experienced considerable sequence divergence. An alternative scenario for the absence of a *Psilanthus* RNase “A” homolog is the subsequent loss of this gene following divergence from the common ancestor with *Coffea.* In the absence of more extensive sampling of *Psilanthus* and other Rubiaceae genera, especially those belonging to Coffeeae and other tribes of subfamily Ixoroideae [70], [71], it is difficult to determine the directionality of gene loss or gain in this case.

Our phylogenetic analysis of RNase T2 genes included a sequence from the species *Hedyotis terminalis* (Rubiaceae) that originated from a publicly available floral EST library [72]. The genus *Hedyotis* is known to contain both SC [73] and GSI species [74], but the biochemical basis of the GSI mechanism is unknown. The placement of this *H. terminalis* RNase in our phylogenetic analysis of RNase T2 genes (see Figure 3) supports the classification by Vieira et al. [17] as a member of the class III subfamily, but amino acid motifs in this sequence are suggestive of an S-like RNase, rather than a functional S-RNase (see below). Supporting this hypothesis, our analysis places the *H. terminalis* RNase in an unresolved position within the class III RNase T2 subfamily with several other S-like RNase genes. While this placement supports the conclusions of Vieira et al. [17], this support is necessarily tentative due to the short length of this sequence in the *H. terminalis* floral EST library.

Vieira et al. [17] surveyed amino acid sequences from a large sample of known S-RNase alleles and S-like genes in order to identify motifs that might be useful in the characterization of RNase genes being mined from rapidly growing whole genome data sets. They found that S-like RNases (i.e. non-S-RNase members of the class III RNase T2 subfamily) generally exhibit an “HEW” amino acid motif directly upstream of the S-RNase conserved region 3 (c3; see Figure 1 and Figure S1, Supporting Information Files). While the *C. arabica* RNase “A” and “C” genes exhibit the HEW motif at this site, this motif also occurs in 7 of the 36 putative *Coffea*/*Psilanthus* S-RNase alleles (i.e. alleles S4, S16, S17, S24, S28, S32, and S37; see Figure 5). Assuming our identification of the *Coffea* S-RNase is accurate, our results suggest that the presence of the HEW amino acid motif may not provide an accurate delimitation of S-like RNases from functional S-RNases, particularly in the absence of gene expression and polymorphism data [17]. Yet, given that the two *Coffea* S-like RNase genes identified in the current study (i.e. RNase “A” and “C”) were found to carry the HEW motif, the possibility of a functional role for this motif in the S-RNase GSI mechanism remains [41], [67].

We have shown that *Coffea* species express three distinct class III RNase T2 genes in pistil tissues and that one of these genes (i.e. the S-RNase) is both polymorphic and heterozygous in SI species of *Coffea* (see Table 1). From the 14 *Coffea* species sampled we have identified 35 putative S-RNase allelic lineages exhibiting considerable sequence divergence (mean pairwise distance = 0.248). Furthermore, a gene tree of putative S-RNase alleles exhibits a clear pattern of trans-specific polymorphism (Figure 5) consistent with the canonical Eudicot S-RNase system. We have shown that the putative *Coffea* S-RNase exhibits expression patterns isolated to pistil tissues (Figure 6), consistent with our observations of the location of the *Coffea* GSI response (Figure 2). Together, these lines of evidence lead to our conclusion that *Coffea* utilizes an S-RNase GSI mechanism that is homologous to the S-RNase based system of GSI that is known to be functional in the three Eudicot families Solanaceae, Plantaginaceae, and Rosaceae. This conclusion is logical given the biochemical complexity of SI mechanisms and the relatively recent common ancestry of Rubiaceae, Solanaceae, and Plantaginaceae within the asterid clade of Eudicots (approximately 90 Ma; [75], [76]). Rubiaceae is thus the fourth Eudicot family to be identified as utilizing the S-RNase GSI system, lending further support for the hypothesis of a single evolutionary origin of this SI mechanism in the ancient ancestral lineage of the Eudicots.

Several questions remain regarding *Coffea* S-RNase GSI. For example, the male determinant S-locus gene (or genes) expressed in pollen tubes has yet to be identified. Furthermore, the mechanism of self-fertility in the diploid species *C. anthonyi* and *C. heterocalyx* remains unclear. While we found no evidence for loss of function mutations in the *C. heterocalyx* S-RNase (S12), previous phylogenetic analyses utilizing samples of this species by Maurin et al. [30] and experiments performed by Coulibaly et al. [38] are a recent hybrid of *C. eugenioides* S.Moore and *C. liberica* Hiern. *var. dewevrei* (De Wild. & T.Durand) Lebrun. Hybridization has been shown to breakdown both the Brassicaceae sporophytic SI mechanism [77] and the Poaceae GSI mechanism [78], but there are also examples of recent hybrid species with fully functioning SI mechanisms [79], [80].

Future work to validate our conclusions might include examining the segregation of S-RNase alleles in mapping populations of *Coffea* trees (see for example [38], [81]). One would expect obligate heterozygosity of S-RNase genotypes in offspring resulting from crosses of known maternal and paternal diploid genotypes. The Rubiaceae contains many genera with heterostylous flowers, and while many of these species exhibit a heteromorphic gametophytic self-incompatibility response [82], the genetic mechanism of this GSI is unknown [83]. By identifying the *Coffea* S-RNase gene, the results presented here are an important foundation for future examinations of Rubiaceae mating systems. For example, a phylogenetic investigation of the distribution of homomorphic and heteromorphic incompatibility systems in the Rubiaceae could be fruitful in identifying the ancestral mating system condition of the family.

## Materials and Methods

### Plant materials and GSI mating system tests

Tissues used in this study were collected from individual plants and small populations of African (6 species sampled) and Madagascan (7 species sampled) *Coffea* maintained in cultivated collections or wild populations in Madagascar, France, and the United States. While all Madagascan species and the majority of African species sampled are thought to be SI [61], three African species (i.e. *C. arabica*, *C. anthonyi*, and *C. heterocalyx*) are known to be SC [29], [38]. Additionally, a single accession of *P. ebracteolatus* was sampled, a self-compatible African species that is closely related to, or perhaps nested within *Coffea* (Davis, unpublished data). Complete information regarding the provenance of each sample used in this study is provided in Table S3 (see Supporting Information Files). Pistil tissues were sampled from each plant in this study and preserved in RNALater (Ambion) for RNA extraction, and formalin–acetic acid–alcohol (FAA) fixative for microscopic analysis.

Each plant from which tissues were collected was also tested for self-incompatibility using a standard aniline blue pollen tube staining procedure adapted from Alexander [66]. This was achieved by covering flowering branches (at least two branches per plant) with a fine nylon mesh 1–2 days before anthesis, returning to the plant when the buds have opened and vigorously shaking the bagged branch to insure self-pollination. After 12–24 hours, the bags were removed and five to ten mature styles were collected for FAA preservation. After 24 hours, FAA preserved styles were transferred to 70% ethanol for storage. The styles were subsequently softened with 1M NaOH, washed repeatedly in water and stained with 10% aniline blue in 0.5M H3PO4 for 24 hours at 4°C. The styles were transferred to a 1:1 mixture of aniline blue stain and glycerol for 4–24 hours at 4°C, and mounted onto glass slides. Gentle pressure applied to the glass cover slip flattened the stained styles tissues, and the slides were viewed through fluorescence microscopy under ultraviolet light with a blue filter.

### Amplification and cloning of *Coffea* RNase T2 genes

Total RNA was extracted from RNALater-preserved styles with the Ambion RNaqueous kit, and RNA concentration was quantified with spectrophotometry (Nanodrop). Due to the level of sequence divergence presumed to exist between any putative *Coffea* RNase T2 genes and those that have been sequenced to date from eudicot species known to utilize the S-RNase system, we employed a standard 3′ RACE (rapid amplification of cDNA ends; [84]) procedure that allowed the use of just one gene-specific degenerate primer to amplify RNase T2 genes from *Coffea* pistil RNA pools. Briefly, first strand cDNA was synthesized from heat-denatured (80°C for 5 mins) total RNA (150 ng – 1 µg) with MMLV reverse transcriptase (Promega), RNase inhibitor (RNaseOut, Invitrogen), and a 3′ RACE adapter primer at 37°C for one hour. The resulting cDNAs were diluted 1:1 with H2O, and used as template in a two-step nested PCR with reverse primers complementary to the 3′ RACE adapter sequence (3′RACE-Outer.R; 3′RACE-Inner.R), and a degenerate forward primer for conserved region c2, which was designed through an analysis of conserved motifs evident between known S-RNase T2 genes [15], [16]. Reactions from the fist step were diluted 1:1 with H2O and employed as template in the second nested PCR. All reactions were performed under standard PCR conditions using Jump Start Taq polymerase (Sigma), and all primer sequences and annealing temperatures are provided in Table S1 (see Supporting Information Files).

Amplicon resulting from the second-step nested PCR was run on a 1% agarose gel stained with 0.25 mg/mL ethidium bromide and all bands larger than approximately 200 bp were extracted from the gel and eluted into 30 uL of sterile water using the UltraClean GelSpin DNA Extraction Kit (Mo-Bio). The resulting gel extracts were cloned using the TOPO TA Kit (Invitrogen) and LB agar plates with 50 µg/mL ampicillin. Approximately 32–48 colonies were screened by PCR (i.e. using the M13 primers provided in the TOPO TA Kit) for insert size and all inserts greater than approximately 200 bp were sequenced with M13 primers at the Duke IGSP DNA Sequencing Facility on an Applied Biosytems 3730 xl capillary sequencer. Preliminary sequencing results allowed the design of less-degenerate forward and reverse primers specific to each of the three *Coffea* RNase T2 gene copies (i.e. “A”, “C”, and S- RNases; see Table S1, Supporting Information Files). Furthermore, several less-degenerate forward/reverse primers for the C2 region of *Coffea* S- RNase T2 genes were designed due to the significant sequence divergence between putative alleles at this locus (see Table S1, Supporting Information Files). Utilizing these gene/allele-specific primers (i.e. in the second step of the nested PCR) provided considerable improvement in the efficiency of RNase T2 amplification from *Coffea* cDNAs. This improved efficiency allowed the characterization of RNase T2 transcripts by screening and sequencing fewer colonies (i.e. from 16–24) from amplicon pools generated using gene/allele-specific primers in nested 3′RACE PCR.

### Analysis of *Coffea* RNase T2 gene expression

We analyzed RNase T2 expression patterns in various tissues of a single *C. arabica* plant growing in the Duke University Greenhouse Teaching Collection (see Table S3, Supporting Information Files). Total RNA was extracted from RNALater-preserved (Ambion) root, fruit, young leaf (i.e.<1 week), mature leaf (i.e.>1 week), petals, and stamens using the methods described above. Following the preliminary cloning of RNase T2 genes described above, we designed primers specific to RNase “A”, “C”, and S-RNase genes known to be expressed in the pistil tissues of this plant (see Table S1, Supporting Information Files), and performed reverse transcriptase PCR and 3′ RACE following the procedures described above. The resulting amplicon was run on a 1.5% agarose gel stained with ethidium bromide and bands were visualized with ultraviolet light.

### Phylogenetic analyses

To test membership in the RNase T2 gene family, we performed a phylogenetic analysis on an amino acid sequence alignment of RNase T2 sequences from Igic and Kohn [15] and several RNase sequences that grouped with Class II and Class III RNase T2 genes in the gene trees published by MacIntosh et al. [41](see Table S2, Supporting Information Files). These RNase T2 genes and *Coffea* RNase T2 genes (non-redundant with respect to putative alleles) were manually aligned using Igic and Kohn's [15] amino acid alignment as a guide. The WAG+I+G model of nucleotide sequence evolution was found to be the best fit to the data using the Akaike Information Criterion in the program ProtTest v.2.4 [85]. Bayesian phylogenetic analyses were performed in triplicate under the best-fit model in the program in MrBayes v3.1.2 [86]. Each run was composed of 4 chains that were sampled every 1000 generations for 10 million generations. Stationarity of each run and convergence of the three chains were verified by examining the lnL scores of all parameters in the program Tracer v1.5 [87]. After removing the first 1 million generations as burn-in, the 50% majority-rule consensus topology and branch lengths were estimated from the 9,001 trees sampled from the posterior distribution. Isoelectric point (pI) estimates were calculated on the ExPASy Compute pI/Mw web server [88]. Pairwise distances were estimated between putative S-RNase allelic lineages using the uncorrected *p* distance with the software PAUP* v.4.10b [89]. Pairwise dN/dS ratio estimates were generated using the maximum likelihood method implemented in the codeml program (i.e. by executing the command “runmode = −2”) within the paml v.4.4 software package [69].

In order to test for trans-specific polymorphism in putative *Coffea* RNase T2 alleles, the gene tree of putative S-RNase alleles was estimated in MrBayes v3.1.2 [86] with model parameters assigned according to the best-fit model (GTR+G+I) estimated in the program MrModeltest v2.0 [90]. The analysis consisted of 3 independent runs, each with 4 chains that were sampled every 1000 generations for 10 million generations. Stationarity of each run and convergence of the three chains were verified by examining the lnL scores of all parameters in the program Tracer v1.5 [87]. After removing the first 1 million generations as burn-in, the 50% majority-rule consensus topology and branch lengths were estimated from the 9,001 trees sampled from the posterior distribution.

## Supporting Information

Figure S1 **Sequence logo for putative** ***Coffea*** **S-RNase alleles.** Sequence logos for each of the *Coffea* RNase T2 genes identified in this study were generated with the WebLogo software [92]. Conserved (i.e. c3–c5) and hyper-variable (i.e. HVa and HVb) regions (see Figure 1) are labeled. The “HEW” motif discussed in the text is labeled in red.
(TIF)

Table S1 **Primer sequences for amplification of Coffea RNase T2 genes.** The primers listed were employed in a standard 3′ RACE procedure and all amplifications used an annealing temperature of 54°C.
(PDF)

Table S2 **Plant RNase T2 sequences employed in phylogenetic analysis shown in** **Figure 3** **.** Genbank accession numbers are provided for all sequences except those from *Glycine max*, but see MacIntosh et al. [41].
(PDF)

Table S3 **Plant materials sampled and sequence accession information.** Sample number refers to the source accession number of individual plants. Genbank sequence accession numbers are provided. Individuals carrying S-RNase alleles identical to other individuals of the same species are indicated by “-” in the sequence accession column.
(PDF)

Table S4 **Pairwise dN/dS Estimates.** Parameter estimates were generated with the maximum likelihood method in the paml software package [69]. A key for pairwise sequence identification is provided at the end of the table.
(PDF)
