# Supplementary figures and images for: Expression and Trans-Specific Polymorphism of Self-Incompatibility RNases in Coffea (Rubiaceae)
Source: PLoS One. 2011 Jun 22;6(6):e21019. doi: 10.1371/journal.pone.0021019 (PMC3120821; doi:10.1371/journal.pone.0021019)

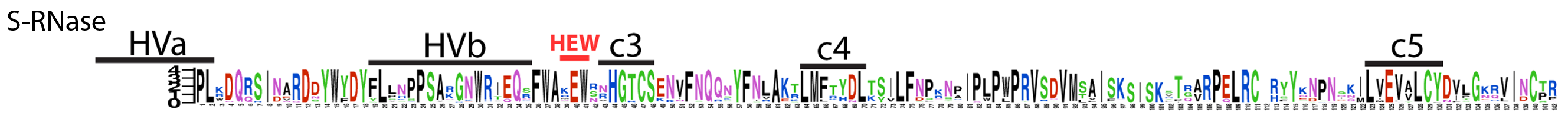

Supplement: Figure S1 — Sequence logo for putative Coffea S-RNase alleles. Sequence logos for each of the Coffea RNase T2 genes identified in this study were generated with the WebLogo software [92]. Conserved (i.e. c3–c5) and hyper-variable (i.e. HVa and HVb) regions (see Figure 1) are labeled. The “HEW” motif discussed in the text is labeled in red. (TIF) [file pone.0021019.s001.tif]
