# Supplementary material for: Expression and Trans-Specific Polymorphism of Self-Incompatibility RNases in Coffea (Rubiaceae)
Source: PLoS One. 2011 Jun 22;6(6):e21019. doi: 10.1371/journal.pone.0021019 (PMC3120821; doi:10.1371/journal.pone.0021019)
Supplement: Table S1 — Primer sequences for amplification of Coffea RNase T2 genes. The primers listed were employed in a standard 3′ RACE procedure and all amplifications used an annealing temperature of 54°C. (PDF) [file pone.0021019.s002.pdf]

Supporting Information Table S1. Primer sequences for amplification of *Coffea* RNase T2 genes.

**Primer Name**	**Sequence (5' - 3')**	**Notes**
Sc2Tot01.F	TTYASIRTYCAYGGIYTRTGGCC	S-RNase Forward primer used in 3'RACE
Sc2Tot03-20bp.F	ASIRTYCAYGGIYTNTGGCC	S-RNase Forward primer used in 3'RACE
CoffBAll-3.F	AAYTRTSGYRGYRSCVSWTAYVKRCC	S-RNase Forward primer used in 3'RACE
CoffBAll-1.R	GYGGAVRWRGVWBDARYABAATCTA	S-RNase Reverse primer used in 3'RACE
Coffc2A-1.F	CAAACCACACTGGYAYAGTGGAAWATTGC	RNase "A" gene specific Forward
CoffendA-1.R	ATACAATCTTGCCGTTTCCTAGYCCTCG	RNase "A" gene specific Reverse
Coffc2C-2.F	CGGGCAAYYTCRCCVAAATTTTGC	RNase "C" gene specific Forward
CoffendC-1.R	CWCCCAGTTCGYAGAAATTGTGAAGGAC	RNase "C" gene specific Reverse
