# Supplementary material for: Expression and Trans-Specific Polymorphism of Self-Incompatibility RNases in Coffea (Rubiaceae)
Source: PLoS One. 2011 Jun 22;6(6):e21019. doi: 10.1371/journal.pone.0021019 (PMC3120821; doi:10.1371/journal.pone.0021019)
Supplement: Table S2 — Plant RNase T2 sequences employed in phylogenetic analysis shown in Figure 3 . Genbank accession numbers are provided for all sequences except those from Glycine max, but see MacIntosh et al. [41]. (PDF) [file pone.0021019.s003.pdf]

Supporting Information Table S2: Plant RNase T2 sequences employed in phylogenetic analysis shown in Figure 3.

Sequence ID	Species	Family	Accession number	Source Publication
Ant.hispanicumS3	*Antirrhinum hispanicum*	Plantaginaceae	AJ315593.1	Vieira et al. (2008)
Ant.mollissSl28	*Antirrhinum mollissimum*	Plantaginaceae	AJ315591.1	Vieira et al. (2008)
Ant.mollissSl29	*Antirrhinum mollissimum*	Plantaginaceae	AJ315592.1	Vieira et al. (2008)
MisopatesSC	*Misopates orontium*	Plantaginaceae	AY013906.1	Vieira et al. (2008)
Ant.hispanicumS5	*Antirrhinum hispanicum*	Plantaginaceae	X96464.1	Igic and Kohn (2001)
Ant.hispanicumS2	*Antirrhinum hispanicum*	Plantaginaceae	AJ300474	Igic and Kohn (2001)
Ant.hispanicumS4	*Antirrhinum hispanicum*	Plantaginaceae	X96466.1	Igic and Kohn (2001)
Pru.aviumS1	*Prunus avium*	Rosaceae	AB028153.1	Igic and Kohn (2001)
Pru.aviumS2	*Prunus avium*	Rosaceae	AB010304.1	Igic and Kohn (2001)
Pru.aviumS3	*Prunus avium*	Rosaceae	AB010306.1	Igic and Kohn (2001)
Pru.aviumS4	*Prunus avium*	Rosaceae	AB028154.1	Igic and Kohn (2001)
Pru.aviumS6	*Prunus avium*	Rosaceae	AB010305.1	Igic and Kohn (2001)
Pru.dulcisPD1	*Prunus dulcis*	Rosaceae	AF227522	Igic and Kohn (2001)
Pru.dulcisPD2	*Prunus dulcis*	Rosaceae	AF202030	Igic and Kohn (2001)
Pru.dulcisSb	*Prunus dulcis*	Rosaceae	AF148466.1	Igic and Kohn (2001)
Pru.dulcisSe	*Prunus dulcis*	Rosaceae	AF177923.1	Vieira et al. (2008)
Pru.salicinaSb	*Prunus saliciana*	Rosaceae	AB026982	Igic and Kohn (2001)
Pru.saliciSa	*Prunus saliciana*	Rosaceae	AB026981	Igic and Kohn (2001)
Pyr.pyrifoliaRNase	*Pyrus pyrifolia*	Rosaceae	D49529	Igic and Kohn (2001)
Pyr.pyrifoliaS1	*Pyrus pyrifolia*	Rosaceae	AB002139.1	Igic and Kohn (2001)
Pyr.pyrifoliaS3	*Pyrus pyrifolia*	Rosaceae	AB025421.1	Igic and Kohn (2001)
Pyr.pyrifoliaS4	*Pyrus pyrifolia*	Rosaceae	AB014072.1	Igic and Kohn (2001)
Pyr.pyrifoliaS5	*Pyrus pyrifolia*	Rosaceae	AB002141.1	Igic and Kohn (2001)
Pyr.pyrifoliaS6	*Pyrus pyrifolia*	Rosaceae	AB002142.1	Igic and Kohn (2001)
Sol.chacoenseS11	*Solanum chacoense*	Solanaceae	S69589	Igic and Kohn (2001)
Sol.chacoenseS12	*Solanum chacoense*	Solanaceae	AF176533.1	Igic and Kohn (2001)
Sol.chacoenseS14	*Solanum chacoense*	Solanaceae	AF232304.1	Igic and Kohn (2001)
Tri.aestivum	*Triticum aestivum*	Poaceae	BF474879	Igic and Kohn (2001)
Vol.carteri	*Volvox carteri*	Volvocaceae	BAA95359.1	Igic and Kohn (2001)

Zea.mays	*Zea mays*	Poaceae	AY107592.1	Vieira et al. (2008)
Zea.maysKIN	*Zea mays*	Poaceae	U66241	Igic and Kohn (2001)
Zin.elegans1	*Zinnia elegans*	Asteraceae	U19923	Igic and Kohn (2001)
Zin.elegans2	*Zinnia elegans*	Asteraceae	U19924	Igic and Kohn (2001)
Ara.thalianaRNS1	*Arabidopsis thaliana*	Brassicaceae	AC004138	Igic and Kohn (2001)
Ara.thalianaRNS2	*Arabidopsis thaliana*	Brassicaceae	AC003000	Igic and Kohn (2001)
Ara.thalianaRNS3	*Arabidopsis thaliana*	Brassicaceae	AC006535	Igic and Kohn (2001)
Ara.thalianaRNS4	*Arabidopsis thaliana*	Brassicaceae	AC007576	Igic and Kohn (2001)
Ara.thalianaRNS5	*Arabidopsis thaliana*	Brassicaceae	AC007576	Igic and Kohn (2001)
Cal.sepium	*Calystegia sepium*	Convolvulaceae	AF139660	Igic and Kohn (2001)
Cic.arietinum	*Cicer arietinum*	Fabaceae	AJ012689	Igic and Kohn (2001)
Gly.max	*Glycine max*	Fabaceae	BG155668	Igic and Kohn (2001)
Gos.hirsutum	*Gossypium hirsutum*	Malvaceae	AI729649	Igic and Kohn (2001)
HetermEST	*Hedyotis terminalis*	Rubiaceae	CB078110.1	Vieira et al. (2008)
Hor.vulgare2	*Hordeum vulgare*	Poaceae	AF000940	Igic and Kohn (2001)
Hor.vulgare4	*Hordeum vulgare*	Poaceae	AL506966	Igic and Kohn (2001)
Hor.vulgarersh1	*Hordeum vulgare*	Poaceae	AF182197	Igic and Kohn (2001)
Hor.vulgareX	*Hordeum vulgare*	Poaceae	BF623030	Igic and Kohn (2001)
Hor.vulgareZ	*Hordeum vulgare*	Poaceae	BE060118	Igic and Kohn (2001)
Luf.cylindricaLC1	*Luffa cylindrica*	Cucurbitaceae	D64012	Igic and Kohn (2001)
Luf.cylindricaLC2	*Luffa cylindrica*	Cucurbitaceae	D64011	Igic and Kohn (2001)
Lyc.esculentumLE	*Lycopersicon esculentum*	Solanaceae	X79337	Igic and Kohn (2001)
Lyc.esculentumLX	*Lycopersicon esculentum*	Solanaceae	AW039494	Igic and Kohn (2001)
Lyc.esculentumRNS2	*Lycopersicon esculentum*	Solanaceae	AK324819.1	Igic and Kohn (2001)
Lyc.peruvianumS11	*Lycium peruvianum*	Solanaceae	U28795.1	Igic and Kohn (2001)
Lyc.peruvianumS12	*Lycium peruvianum*	Solanaceae	U28796.1	Igic and Kohn (2001)
Lyc.peruvianumS3	*Lycium peruvianum*	Solanaceae	X76065.1	Igic and Kohn (2001)
Mal.domesticaSd	*Malus domestica*	Rosaceae	AB032246	Igic and Kohn (2001)
Mal.domesticaSe	*Malus domestica*	Rosaceae	AB035273.1	Igic and Kohn (2001)
Med.truncatula2	*Medicago truncatula*	Fabaceae	BF645898	Igic and Kohn (2001)
Med.truncatula4	*Medicago truncatula*	Fabaceae	BG455183	Igic and Kohn (2001)

Med.truncatula5	*Medicago truncatula*	Fabaceae	BG451455	Igic and Kohn (2001)
N.alataSA2	*Nicotiana alata*	Solanaceae	U08860.1	Igic and Kohn (2001)
Nel.nucifera	*Nelumbo nucifera*	Nelumbonaceae	M83668.1	Igic and Kohn (2001)
Nic.alataNE	*Nicotiana alata*	Solanaceae	U13256.1	Igic and Kohn (2001)
Nic.alataS2	*Nicotiana alata*	Solanaceae	U08860.1	Igic and Kohn (2001)
Nic.glutinosaNGR2	*Nicotiana glutinosa*	Solanaceae	AB032256	Igic and Kohn (2001)
Nic.glutinosaNGR3	*Nicotiana glutinosa*	Solanaceae	AB032257	Igic and Kohn (2001)
Nic.tabacumNK1	*Nicotiana tabacum*	Solanaceae	AB034638	Igic and Kohn (2001)
Ory.sativa1	*Oryza sativa*	Poaceae	AB052842	Igic and Kohn (2001)
Ory.sativa2	*Oryza sativa*	Poaceae	AU091979	Igic and Kohn (2001)
Pin.taeda	*Pinus taeda*	Pinaceae	BF186081	Igic and Kohn (2001)
Pis.sativumHGRP	*Pisum sativum*	Fabaceae	Y11824.1	Igic and Kohn (2001)
GmaRNS12	*Glycine max*	Fabaceae	Glyma03g35230	MacIntosh et al. (2010)
GmaRNS5	*Glycine max*	Fabaceae	Glyma02g07130	MacIntosh et al. (2010)
GmaRNS4	*Glycine max*	Fabaceae	Glyma02g07140	MacIntosh et al. (2010)
GmaRNS3	*Glycine max*	Fabaceae	Glyma02g07150	MacIntosh et al. (2010)
GmaRNS6	*Glycine max*	Fabaceae	Glyma07g06520	MacIntosh et al. (2010)
GmaRNS2	*Glycine max*	Fabaceae	Glyma16g03120	MacIntosh et al. (2010)
RnaseLER	*Solanum lycopersicum*	Solanaceae	CAL64053	MacIntosh et al. (2010)
CAC50874	*Antirrhinum hispanicum*	Plantaginaceae	CAC50874	MacIntosh et al. (2010)
PtrRNS2	*Populus trichocarpa*	Betulaceae	XP_002321228	MacIntosh et al. (2010)
Miguf021969m	*Mimulus guttatus*	Scrophulariaceae	mgf021969m	Phytozome v.5.0
PtrRNS5	*Populus trichocarpa*	Betulaceae	EEE95823	MacIntosh et al. (2010)
