# Supplementary material for: Expression and Trans-Specific Polymorphism of Self-Incompatibility RNases in Coffea (Rubiaceae)
Source: PLoS One. 2011 Jun 22;6(6):e21019. doi: 10.1371/journal.pone.0021019 (PMC3120821; doi:10.1371/journal.pone.0021019)
Supplement: Table S3 — Plant materials sampled and sequence accession information. Sample number refers to the source accession number of individual plants. Genbank sequence accession numbers are provided. Individuals carrying S-RNase alleles identical to other individuals of the same species are indicated by “-” in the sequence accession column. (PDF) [file pone.0021019.s004.pdf]

RNA#	Species	Sample Number	Native Distribution	Source	Sequence Accession Numbers
MNR69	C. andrambovatensis	A227-1	Madagascar	Kianjavato Station, Madagascar	JN035340; JN035341; JN035342
MNR70	C. andrambovatensis	A310-1	Madagascar	Kianjavato Station, Madagascar	JN035343
MNR71	C. andrambovatensis	A310-2	Madagascar	Kianjavato Station, Madagascar	JN035344
MNR72	C. andrambovatensis	A310-3	Madagascar	Kianjavato Station, Madagascar	JN035345
MNR80	C. bonnieri	A535-2	Madagascar	Kianjavato Station, Madagascar	JN035353; JN035356
MNR81	C. bonnieri	A535-3	Madagascar	Kianjavato Station, Madagascar	JN035354
MNR82	C. bonnieri	A535-1	Madagascar	Kianjavato Station, Madagascar	JN035352; JN035355
MNR106	C. millotii	A572-1	Madagascar	Kianjavato Station, Madagascar	-
MNR107	C. millotii	A572-2	Madagascar	Kianjavato Station, Madagascar	-
MNR61	C. millotii	A206-1	Madagascar	Kianjavato Station, Madagascar	JN035337
MNR62	C. millotii	A206-2	Madagascar	Kianjavato Station, Madagascar	JN035334; JN035338
MNR63	C. millotii	A206-3	Madagascar	Kianjavato Station, Madagascar	-
MNR64	C. millotii	A206-4	Madagascar	Kianjavato Station, Madagascar	JN035335
MNR65	C. millotii	A311-1	Madagascar	Kianjavato Station, Madagascar	JN035339
MNR66	C. millotii	A311-3	Madagascar	Kianjavato Station, Madagascar	-
MNR67	C. millotii	A311-4	Madagascar	Kianjavato Station, Madagascar	JN035336
MNR68	C. millotii	A311-5	Madagascar	Kianjavato Station, Madagascar	JN035333
MNR76	C. montis-sacri	A321-1	Madagascar	Kianjavato Station, Madagascar	JN035348; JN035349
MNR77	C. montis-sacri	A321-2	Madagascar	Kianjavato Station, Madagascar	JN035346; JN035351
MNR78	C. montis-sacri	A321-3	Madagascar	Kianjavato Station, Madagascar	JN035347; JN035350
MNR79	C. montis-sacri	A321-4	Madagascar	Kianjavato Station, Madagascar	-
MNR46B	C. perrieri	A12-1	Madagascar	Kianjavato Station, Madagascar	-
MNR47	C. perrieri	A12-2	Madagascar	Kianjavato Station, Madagascar	JN035327
MNR48	C. perrieri	A12-3	Madagascar	Kianjavato Station, Madagascar	-
MNR49	C. perrieri	A12-4	Madagascar	Kianjavato Station, Madagascar	-
MNR50	C. perrieri	A12-5	Madagascar	Kianjavato Station, Madagascar	-
MNR51	C. perrieri	A12-6	Madagascar	Kianjavato Station, Madagascar	-
MNR52	C. perrieri	A12-7	Madagascar	Kianjavato Station, Madagascar	JN035328
MNR53	C. perrieri	A12-8	Madagascar	Kianjavato Station, Madagascar	JN035329
MNR54	C. perrieri	A12-9	Madagascar	Kianjavato Station, Madagascar	JN035330
MNR59	C. perrieri	A421-1	Madagascar	Kianjavato Station, Madagascar	JN035331
MNR60	C. perrieri	A421-2	Madagascar	Kianjavato Station, Madagascar	JN035332
MNR89	C. resinosa	A841-7	Madagascar	Kianjavato Station, Madagascar	-
MNR90	C. resinosa	A841-4	Madagascar	Kianjavato Station, Madagascar	JN035360
MNR91	C. resinosa	A841-2	Madagascar	Kianjavato Station, Madagascar	JN035366
MNR92	C. resinosa	A841-6	Madagascar	Kianjavato Station, Madagascar	JN035364
MNR93	C. resinosa	A841-1	Madagascar	Kianjavato Station, Madagascar	-
MNR94	C. resinosa	A841-3	Madagascar	Kianjavato Station, Madagascar	JN035361; JN035363; JN035365
MNR95	C. resinosa	A841-4	Madagascar	Kianjavato Station, Madagascar	JN035362
MNR83	C. tricalysoides	A730-1	Madagascar	Kianjavato Station, Madagascar	JN035357
MNR84	C. tricalysoides	A730-2	Madagascar	Kianjavato Station, Madagascar	JN035358; JN035359
MNR35	C. tsirinanae	MN19F	Madagascar	Mt. des Francais, Madagascar	JN035315
MNR36	C. tsirinanae	MN19L	Madagascar	Mt. des Francais, Madagascar	JN035316; JN035318
MNR37	C. tsirinanae	MN20G	Madagascar	Mt. des Francais, Madagascar	JN035317
MNR38	C. tsirinanae	MN21A	Madagascar	Mt. des Francais, Madagascar	-
MNR39	C. tsirinanae	MN24A	Madagascar	Mt. des Francais, Madagascar	JN035319; JN035320
MNR46A	C. arabica		Africa	Duke	JN035321; JN035322; JN035323
MNR123	C. brevipes	JB68	Africa	Montpellier	JN035313
MNR02	C. canephora	94535c	Africa	Fairchild Gardens	JN035305
MNR118	C. canephora	BC51	Africa	Montpellier	JN035306; JN035307
MNR119	C. canephora	BC62	Africa	Montpellier	JN035308; JN035309
MNR55	C. eugenoides	A16-G25	Africa	Kianjavato Station, Madagascar	JN035324
MNR56	C. eugenoides	A16-G23	Africa	Kianjavato Station, Madagascar	JN035325
MNR57	C. eugenoides	A16-G24	Africa	Kianjavato Station, Madagascar	JN035326
MNR58	C. eugenoides	A16-G17	Africa	Kianjavato Station, Madagascar	-
MNR124	C. heterocalyx	JC65	Africa	Montpellier	JN035314
MNR125	C. heterocalyx	JC63	Africa	Montpellier	-
MNR126	C. heterocalyx	JC61	Africa	Montpellier	-
MNR127	C. heterocalyx	JC62	Africa	Montpellier	-
MNR121	C. pseudozanguebariae	H60	Africa	Montpellier	JN035310; JN035311
MNR122	Psilanthus ebracteolatus	PAE no.2	Africa	Montpellier	JN035312
