# Supplementary material for: Expression and Trans-Specific Polymorphism of Self-Incompatibility RNases in Coffea (Rubiaceae)
Source: PLoS One. 2011 Jun 22;6(6):e21019. doi: 10.1371/journal.pone.0021019 (PMC3120821; doi:10.1371/journal.pone.0021019)
Supplement: Table S4 — Pairwise dN/dS Estimates. Parameter estimates were generated with the maximum likelihood method in the paml software package [69]. A key for pairwise sequence identification is provided at the end of the table. (PDF) [file pone.0021019.s005.pdf]

Table S4. Pairwise dN/dS Estimates.

Summary Statistics:

Mean dN/dS	StDev dN/dS	Min dN/dS	Max dN/dS
0.361727937	0.096725808	0.1531	0.8719

seq	seq	N	S	dN	dS	dN/dS	Paras.			
2	1	313	107	0.2135	0.5073	0.4209	0.8652	2.6745	0.4209	-864.922
3	1	306.3	113.7	0.1858	0.4968	0.3739	0.8099	3.2165	0.3739	-848.715
3	2	312.4	107.6	0.1861	0.3811	0.4884	0.7082	2.5902	0.4884	-840.923
4	1	304.7	115.3	0.1918	0.4721	0.4062	0.8061	2.985	0.4062	-855.951
4	2	308.8	111.2	0.206	0.508	0.4055	0.8579	2.4823	0.4055	-873.912
4	3	302.7	117.3	0.2254	0.4897	0.4604	0.8977	3.1917	0.4604	-877.68
5	1	317	103	0.1515	0.742	0.2042	0.8888	1.999	0.2042	-846.033
5	2	315.3	104.7	0.1907	0.4392	0.4341	0.7578	2.2201	0.4341	-849.679
5	3	306.7	113.3	0.1885	0.5111	0.3687	0.8265	3.0439	0.3687	-851.032
5	4	317.7	102.3	0.1803	0.58	0.3109	0.8331	1.5884	0.3109	-868.661
6	1	307.1	112.9	0.1795	0.4427	0.4054	0.7507	2.9219	0.4054	-843.482
6	2	309.5	110.5	0.1792	0.426	0.4206	0.7323	2.9082	0.4206	-840.017
6	3	307.8	112.2	0.1881	0.5167	0.364	0.8276	2.8636	0.364	-858.484
6	4	306	114	0.1647	0.6432	0.2561	0.8838	2.506	0.2561	-858.944
6	5	315	105	0.1794	0.7643	0.2348	0.977	1.9902	0.2348	-867.89
7	1	308.6	111.4	0.1967	0.5114	0.3846	0.8405	2.9474	0.3846	-858.197
7	2	314.9	105.1	0.1417	0.4368	0.3244	0.6466	2.2851	0.3244	-825.865
7	3	307.8	112.2	0.2017	0.4026	0.501	0.766	2.9272	0.501	-853.844
7	4	310.8	109.2	0.1564	0.4737	0.3303	0.7168	2.069	0.3303	-845.797
7	5	319.7	100.3	0.1478	0.5712	0.2587	0.7466	1.7066	0.2587	-839.424
7	6	310.7	109.3	0.1317	0.5074	0.2595	0.6884	2.4078	0.2595	-822.296
8	1	316.3	103.7	0.1567	0.652	0.2403	0.8368	1.9953	0.2403	-850.624
8	2	315.3	104.7	0.204	0.4079	0.5001	0.7645	2.1505	0.5001	-859.162
8	3	312.7	107.3	0.1992	0.5559	0.3583	0.871	2.2481	0.3583	-870.648
8	4	311.7	108.3	0.1845	0.6544	0.282	0.9171	2.0167	0.282	-875.54
8	5	321	99	0.1699	0.6556	0.2591	0.8531	1.6283	0.2591	-857.173
8	6	313	107	0.1671	0.5367	0.3113	0.7835	2.1218	0.3113	-849.043
8	7	309.4	110.6	0.1745	0.4078	0.4278	0.7078	2.6464	0.4278	-841.565
9	1	310	110	0.1478	0.5998	0.2465	0.7987	2.6083	0.2465	-833.509
9	2	308.6	111.4	0.1629	0.6078	0.268	0.8427	3.0055	0.268	-843.331
9	3	304.5	115.5	0.1928	0.4769	0.4043	0.8128	3.4473	0.4043	-848.477
9	4	306.8	113.2	0.1959	0.6719	0.2916	0.9727	2.5745	0.2916	-879.263
9	5	309.3	110.7	0.145	0.7672	0.189	0.927	2.6723	0.189	-837.075
9	6	307.3	112.7	0.1531	0.6103	0.2509	0.8275	2.9778	0.2509	-835.816
9	7	302.2	117.8	0.1303	0.5959	0.2187	0.7829	3.8615	0.2187	-816.81
9	8	310.7	109.3	0.1757	0.6309	0.2785	0.8823	2.4519	0.2785	-854.12
10	1	316	104	0.1484	0.4694	0.3162	0.6836	2.1672	0.3162	-825.98
10	2	317.2	102.8	0.1839	0.5339	0.3444	0.8088	2.2191	0.3444	-856.189
10	3	312.4	107.6	0.164	0.4734	0.3464	0.7297	2.432	0.3464	-839.369
10	4	310.5	109.5	0.191	0.5008	0.3815	0.8153	2.198	0.3815	-867.02
10	5	316.5	103.5	0.1541	0.5786	0.2663	0.7759	2.0311	0.2663	-841.349
10	6	309.1	110.9	0.1855	0.4551	0.4077	0.7701	2.7541	0.4077	-847.587
10	7	313.1	106.9	0.1817	0.5149	0.3528	0.7995	2.3795	0.3528	-857.295
10	8	321.4	98.6	0.1613	0.4766	0.3384	0.7059	1.5717	0.3384	-844.067
10	9	312.5	107.5	0.1613	0.6104	0.2643	0.8288	2.354	0.2643	-845.327
11	1	307.7	112.3	0.1779	0.5401	0.3293	0.8243	3.1684	0.3293	-845.938
11	2	313.1	106.9	0.2177	0.5223	0.4168	0.8857	2.6768	0.4168	-871.775
11	3	310.6	109.4	0.1991	0.4246	0.4689	0.7734	2.5429	0.4689	-858.967
11	4	310.2	109.8	0.194	0.5657	0.3429	0.8734	2.0815	0.3429	-875.393
11	5	310	110	0.196	0.5392	0.3635	0.8577	2.6342	0.3635	-863.905
11	6	309.5	110.5	0.165	0.5767	0.2861	0.82	2.5659	0.2861	-848.879
11	7	309.6	110.4	0.1914	0.5146	0.3718	0.8291	2.7508	0.3718	-858.567
11	8	313.5	106.5	0.1876	0.4847	0.387	0.7887	2.1655	0.387	-859.283
11	9	312.9	107.1	0.1922	0.5725	0.3358	0.8676	2.219	0.3358	-865.213
11	10	312.5	107.5	0.201	0.4733	0.4246	0.812	2.5397	0.4246	-860.652
12	1	304.3	115.7	0.1967	0.513	0.3835	0.8515	3.0959	0.3835	-858.75
12	2	310.1	109.9	0.2259	0.497	0.4546	0.8906	2.4672	0.4546	-880.913

12	3	301.4	118.6	0.2408	0.5702	0.4222	1.0015	3.6002	0.4222	-887.407
12	4	304.9	115.1	0.178	0.558	0.319	0.8466	2.4925	0.319	-864.231
12	5	314.7	105.3	0.1914	0.5919	0.3233	0.8754	1.9165	0.3233	-868.492
12	6	302.7	117.3	0.2056	0.5808	0.3539	0.931	3.3006	0.3539	-868.955
12	7	304.3	115.7	0.1916	0.5337	0.359	0.8576	3.08	0.359	-863.547
12	8	310.8	109.2	0.1921	0.4791	0.401	0.8002	2.221	0.401	-861.85
12	9	303.5	116.5	0.1889	0.5618	0.3363	0.877	3.2377	0.3363	-859.328
12	10	304.7	115.3	0.1766	0.5395	0.3274	0.8287	3.0921	0.3274	-851.912
12	11	306.1	113.9	0.207	0.5799	0.3569	0.9244	2.7455	0.3569	-876.934
13	1	324.3	95.7	0.1815	0.5434	0.3339	0.7918	1.526	0.3339	-854.05
13	2	317.5	102.5	0.1767	0.3403	0.5192	0.6498	2.3243	0.5192	-829.703
13	3	317.4	102.6	0.1635	0.4557	0.3587	0.7045	1.9908	0.3587	-839.841
13	4	316.5	103.5	0.1877	0.4829	0.3887	0.7813	1.6485	0.3887	-865.492
13	5	324.3	95.7	0.1861	0.5975	0.3114	0.8395	1.4801	0.3114	-862.82
13	6	324.6	95.4	0.1282	0.5212	0.246	0.6524	1.2853	0.246	-817.361
13	7	318.3	101.7	0.1265	0.4865	0.2599	0.641	1.9203	0.2599	-816.725
13	8	324.1	95.9	0.1785	0.4976	0.3587	0.7541	1.4227	0.3587	-854.755
13	9	314.1	105.9	0.1555	0.5629	0.2763	0.7746	2.304	0.2763	-834.006
13	10	327.1	92.9	0.1551	0.5816	0.2667	0.7483	1.3271	0.2667	-840.837
13	11	319.4	100.6	0.1785	0.503	0.3548	0.7687	1.8763	0.3548	-850.461
13	12	315.2	104.8	0.2033	0.5708	0.3561	0.8849	1.9232	0.3561	-875.893
14	1	309.4	110.6	0.2402	0.3765	0.6379	0.8283	2.5844	0.6379	-866.388
14	2	312.4	107.6	0.2253	0.4143	0.5438	0.8211	2.4384	0.5438	-868.654
14	3	308.6	111.4	0.2203	0.4565	0.4826	0.8488	2.5906	0.4826	-873.17
14	4	304.9	115.1	0.2395	0.5171	0.4631	0.9466	2.5632	0.4631	-892.087
14	5	318.3	101.7	0.2266	0.6601	0.3433	0.9947	1.6968	0.3433	-890.482
14	6	309.9	110.1	0.2047	0.4729	0.4328	0.825	2.344	0.4328	-865.574
14	7	308.6	111.4	0.2151	0.4607	0.4668	0.8406	2.6106	0.4668	-868.368
14	8	316.2	103.8	0.2281	0.4739	0.4813	0.8666	1.7946	0.4813	-879.879
14	9	308.8	111.2	0.247	0.4779	0.5168	0.9245	2.5486	0.5168	-883.895
14	10	314.8	105.2	0.2402	0.4369	0.5498	0.8684	2.0287	0.5498	-881.65
14	11	307.2	112.8	0.2452	0.515	0.4761	0.953	2.8501	0.4761	-885.934
14	12	307	113	0.2408	0.4585	0.5253	0.8982	2.4278	0.5253	-885.519
14	13	315.3	104.7	0.2163	0.4758	0.4545	0.8428	2.0977	0.4545	-872.242
15	1	308.6	111.4	0.1804	0.6918	0.2607	0.948	2.6074	0.2607	-864.072
15	2	314.8	105.2	0.2095	0.4066	0.5153	0.7766	2.0356	0.5153	-863.152
15	3	309	111	0.2297	0.5159	0.4452	0.916	2.4763	0.4452	-885.064
15	4	310.3	109.7	0.1501	0.7334	0.2047	0.9072	1.896	0.2047	-859.313
15	5	314.4	105.6	0.1899	0.7762	0.2447	1.0121	1.9863	0.2447	-877.832
15	6	311.7	108.3	0.1992	0.5519	0.3609	0.8704	2.0874	0.3609	-870.74
15	7	313	107	0.2023	0.5488	0.3687	0.8718	2.0314	0.3687	-876.446
15	8	314.3	105.7	0.2058	0.4501	0.4572	0.8018	1.9234	0.4572	-868.594
15	9	310.2	109.8	0.2183	0.6858	0.3184	1.0216	2.3617	0.3184	-887.161
15	10	313.3	106.7	0.2111	0.5498	0.384	0.8915	2.1008	0.384	-879.857
15	11	313.7	106.3	0.1661	0.5066	0.3279	0.7568	1.924	0.3279	-850.539
15	12	308	112	0.1712	0.5377	0.3183	0.8069	2.3115	0.3183	-858.669
15	13	321.2	98.8	0.2025	0.502	0.4035	0.8191	1.4768	0.4035	-870.812
15	14	310.4	109.6	0.204	0.5924	0.3444	0.9162	2.1355	0.3444	-876.452
16	1	306.3	113.7	0.209	0.4121	0.5073	0.7921	3.1074	0.5073	-857.521
16	2	307.5	112.5	0.2051	0.4891	0.4194	0.8436	3.2273	0.4194	-861.935
16	3	303.5	116.5	0.1957	0.4479	0.437	0.797	3.4884	0.437	-854.546
16	4	306.5	113.5	0.1593	0.5412	0.2944	0.7875	2.3756	0.2944	-852.074
16	5	310.4	109.6	0.158	0.6954	0.2272	0.8947	2.4426	0.2272	-852.068
16	6	307.6	112.4	0.1431	0.4346	0.3292	0.6633	2.6385	0.3292	-822.641
16	7	305.1	114.9	0.1789	0.4259	0.4202	0.7396	3.2003	0.4202	-845.098
16	8	313.3	106.7	0.1789	0.5156	0.347	0.7933	2.0445	0.347	-860.673
16	9	302.7	117.3	0.2041	0.5338	0.3824	0.8886	3.4886	0.3824	-864.376
16	10	312.3	107.7	0.166	0.4386	0.3785	0.7077	2.3325	0.3785	-840.736
16	11	303.7	116.3	0.1748	0.502	0.3482	0.7963	3.5811	0.3482	-847.258
16	12	301.9	118.1	0.1939	0.5254	0.3691	0.8614	3.245	0.3691	-865.144
16	13	317.3	102.7	0.1743	0.4448	0.3918	0.7214	1.9032	0.3918	-845.312
16	14	304.2	115.8	0.2316	0.4707	0.492	0.8926	3.057	0.492	-878.67
16	15	311.7	108.3	0.1632	0.5932	0.2751	0.8224	2.0013	0.2751	-858.343
17	1	314.4	105.6	0.1463	0.5438	0.2691	0.7388	2.2953	0.2691	-828.406

17	2	318.9	101.1	0.1586	0.5632	0.2816	0.7679	2.0497	0.2816	-838.089
17	3	312.9	107.1	0.1656	0.5765	0.2873	0.8112	2.4084	0.2873	-847.877
17	4	313.7	106.3	0.1699	0.63	0.2696	0.859	1.8552	0.2696	-863.224
17	5	317.1	102.9	0.1396	0.7071	0.1974	0.8361	1.9766	0.1974	-835.074
17	6	317.3	102.7	0.1646	0.5806	0.2836	0.799	1.8786	0.2836	-847.887
17	7	310	110	0.1596	0.5109	0.3123	0.7547	2.8066	0.3123	-838.01
17	8	317.3	102.7	0.1491	0.7076	0.2107	0.857	1.9105	0.2107	-846.857
17	9	309.4	110.6	0.1507	0.8127	0.1854	0.975	2.8491	0.1854	-843.151
17	10	314.9	105.1	0.1618	0.554	0.292	0.7797	2.3211	0.292	-843.328
17	11	314.8	105.2	0.1966	0.6111	0.3217	0.9011	2.2361	0.3217	-866.524
17	12	311.4	108.6	0.1954	0.4118	0.4744	0.7539	2.2425	0.4744	-856.25
17	13	323.2	96.8	0.1664	0.7837	0.2123	0.926	1.6401	0.2123	-858.756
17	14	315.2	104.8	0.2129	0.4173	0.5101	0.7917	2.012	0.5101	-860.5
17	15	318.3	101.7	0.166	0.6697	0.2479	0.8639	1.6538	0.2479	-857.142
17	16	313.9	106.1	0.1854	0.581	0.3192	0.8562	2.185	0.3192	-864.084
18	1	304.4	115.6	0.1871	0.415	0.4508	0.7495	3.5548	0.4508	-840.012
18	2	302.1	117.9	0.1896	0.4058	0.4672	0.7508	4.5914	0.4672	-837.238
18	3	299.6	120.4	0.1868	0.3442	0.5425	0.6957	4.5062	0.5425	-830.947
18	4	300.7	119.3	0.1977	0.4392	0.4503	0.799	3.3522	0.4503	-860.413
18	5	309.4	110.6	0.1867	0.395	0.4725	0.7247	2.5878	0.4725	-848.765
18	6	300.1	119.9	0.1907	0.4735	0.4027	0.8143	4.0863	0.4027	-849.626
18	7	300.6	119.4	0.1967	0.416	0.4728	0.7771	4.2468	0.4728	-848.248
18	8	310.1	109.9	0.1965	0.4621	0.4252	0.798	2.4071	0.4252	-862.353
18	9	304.8	115.2	0.1609	0.5261	0.3059	0.7834	3.1364	0.3059	-838.44
18	10	307.9	112.1	0.1728	0.3374	0.5123	0.6502	3.0033	0.5123	-829.015
18	11	306.2	113.8	0.1732	0.458	0.3783	0.7512	3.2324	0.3783	-841.474
18	12	296.7	123.3	0.253	0.436	0.5803	0.9201	4.4178	0.5803	-878.751
18	13	311.2	108.8	0.176	0.4696	0.3748	0.7561	2.6679	0.3748	-847.029
18	14	298.6	121.4	0.2615	0.4007	0.6524	0.9052	4.3011	0.6524	-876.311
18	15	304.3	115.7	0.2181	0.4253	0.5127	0.8256	3.0124	0.5127	-869.388
18	16	299.1	120.9	0.2248	0.4291	0.5239	0.8507	4.2664	0.5239	-865.93
18	17	306.9	113.1	0.1726	0.5468	0.3156	0.82	3.2302	0.3156	-846.832
19	1	306.5	113.5	0.2008	0.4184	0.4799	0.7788	3.1347	0.4799	-849.613
19	2	308	112	0.253	0.4355	0.5809	0.9049	3.1835	0.5809	-878.726
19	3	304.8	115.2	0.2613	0.3711	0.7041	0.8743	3.2963	0.7041	-878.626
19	4	307.5	112.5	0.173	0.4746	0.3645	0.7613	2.3389	0.3645	-852.208
19	5	308.4	111.6	0.2069	0.5536	0.3738	0.8971	2.7312	0.3738	-868.898
19	6	306.7	113.3	0.1989	0.4892	0.4065	0.8316	2.8316	0.4065	-861.118
19	7	306.2	113.8	0.199	0.4135	0.4814	0.7714	3.0816	0.4814	-854.615
19	8	308.4	111.6	0.2347	0.4133	0.5679	0.8464	2.6526	0.5679	-874.29
19	9	304.3	115.7	0.2256	0.6118	0.3687	0.9959	3.2849	0.3687	-879.468
19	10	313.6	106.4	0.2329	0.3674	0.6339	0.8009	2.2129	0.6339	-870.068
19	11	305.4	114.6	0.2339	0.4163	0.5618	0.851	3.2923	0.5618	-870.361
19	12	301.6	118.4	0.2252	0.3997	0.5634	0.8232	3.3871	0.5634	-867.449
19	13	312.3	107.7	0.2387	0.5124	0.4659	0.9267	2.4973	0.4659	-881.423
19	14	306.2	113.8	0.3103	0.3559	0.8719	0.9681	2.8145	0.8719	-900.133
19	15	307.7	112.3	0.2102	0.5071	0.4145	0.8687	2.5329	0.4145	-871.461
19	16	307.3	112.7	0.1871	0.4158	0.4501	0.7455	2.7007	0.4501	-852.07
19	17	316.1	103.9	0.1816	0.4301	0.4222	0.7291	1.9529	0.4222	-847.144
19	18	303.6	116.4	0.262	0.3438	0.7622	0.8542	3.4028	0.7622	-875.031
20	1	314.2	105.8	0.2122	0.5553	0.3821	0.8959	1.8867	0.3821	-881.857
20	2	319	101	0.2112	0.5578	0.3786	0.8836	1.5242	0.3786	-884.065
20	3	314.7	105.3	0.1877	0.5688	0.33	0.8497	1.7031	0.33	-874.882
20	4	309.1	110.9	0.1933	0.5802	0.3332	0.8862	1.8166	0.3332	-881.471
20	5	318.8	101.2	0.2001	0.9195	0.2177	1.1206	1.5032	0.2177	-896.931
20	6	312.2	107.8	0.1594	0.5772	0.2761	0.7998	1.8497	0.2761	-850.258
20	7	315.5	104.5	0.1766	0.6002	0.2942	0.8458	1.6487	0.2942	-870.38
20	8	317.6	102.4	0.1997	0.6196	0.3224	0.9063	1.5089	0.3224	-884.66
20	9	312.4	107.6	0.2074	0.5603	0.3701	0.8933	1.9529	0.3701	-877.609
20	10	313.9	106.1	0.2029	0.5532	0.3668	0.8743	1.9206	0.3668	-879.02
20	11	315	105	0.1756	0.657	0.2673	0.8878	1.6748	0.2673	-871.708
20	12	308.2	111.8	0.2036	0.5789	0.3517	0.9103	2.0808	0.3517	-883.951
20	13	327	93	0.1704	0.4698	0.3627	0.7102	0.9775	0.3627	-851.39
20	14	310	110	0.2032	0.3778	0.538	0.7468	2.021	0.538	-860.188

20	15	314.6	105.4	0.1786	0.6531	0.2735	0.8932	1.5227	0.2735	-874.233
20	16	308.9	111.1	0.1822	0.5451	0.3342	0.8345	2.1488	0.3342	-866.836
20	17	316.9	103.1	0.1718	0.5853	0.2936	0.82	1.66	0.2936	-859.099
20	18	305.1	114.9	0.2193	0.5603	0.3914	0.9377	2.6979	0.3914	-886.756
20	19	312.7	107.3	0.2183	0.5552	0.3932	0.9131	1.7981	0.3932	-890.704
21	1	319.7	100.3	0.1382	0.4884	0.283	0.6655	2.1013	0.283	-819.463
21	2	324.8	95.2	0.1798	0.5225	0.3442	0.7725	1.7722	0.3442	-855.032
21	3	317.8	102.2	0.1746	0.4522	0.3861	0.7264	2.1257	0.3861	-845.633
21	4	315.2	104.8	0.15	0.4711	0.3184	0.6903	1.975	0.3184	-837.899
21	5	331.8	88.2	0.1077	0.7033	0.1531	0.6983	1.1392	0.1531	-815.299
21	6	312	108	0.1502	0.5753	0.2611	0.7785	2.7877	0.2611	-832.847
21	7	317.3	102.7	0.1167	0.4962	0.2352	0.6284	2.2284	0.2352	-806.707
21	8	319.4	100.6	0.1555	0.5864	0.2652	0.7761	1.9506	0.2652	-845.792
21	9	311.8	108.2	0.1351	0.7467	0.1809	0.8782	2.8027	0.1809	-828.815
21	10	327.3	92.7	0.1439	0.5834	0.2466	0.7228	1.4759	0.2466	-836.65
21	11	322.8	97.2	0.1635	0.4709	0.3472	0.7039	1.7518	0.3472	-839.875
21	12	312.2	107.8	0.1765	0.6136	0.2877	0.8662	2.4343	0.2877	-861.098
21	13	338.3	81.7	0.139	0.5435	0.2557	0.6529	0.8251	0.2557	-823.952
21	14	322.5	97.5	0.2175	0.5363	0.4056	0.8746	1.6199	0.4056	-879.63
21	15	319.8	100.2	0.1752	0.5979	0.2931	0.8283	1.7511	0.2931	-863.202
21	16	318.3	101.7	0.152	0.4988	0.3047	0.7079	2.0311	0.3047	-838.368
21	17	323.5	96.5	0.1479	0.6151	0.2405	0.7657	1.7547	0.2405	-837.74
21	18	312.1	107.9	0.1858	0.4019	0.4624	0.7241	2.9061	0.4624	-846.991
21	19	314	106	0.2059	0.4638	0.4439	0.8129	2.5433	0.4439	-861.049
21	20	324.8	95.2	0.1671	0.5171	0.3231	0.7393	1.2661	0.3231	-850.712
22	1	317.3	102.7	0.1702	0.4781	0.356	0.7364	1.8777	0.356	-842.579
22	2	315.4	104.6	0.1607	0.4637	0.3466	0.7086	2.1659	0.3466	-835.558
22	3	307.7	112.3	0.149	0.4079	0.3652	0.6546	2.8553	0.3652	-819.486
22	4	313	107	0.1925	0.5024	0.3831	0.8143	1.8253	0.3831	-871.642
22	5	320.4	99.6	0.1681	0.5728	0.2935	0.7924	1.6299	0.2935	-850.241
22	6	314.6	105.4	0.1491	0.4097	0.3638	0.6434	1.9616	0.3638	-825.015
22	7	307.6	112.4	0.1325	0.3802	0.3484	0.5964	2.8791	0.3484	-804.794
22	8	308.6	111.4	0.1872	0.4388	0.4266	0.7617	2.6588	0.4266	-847.2
22	9	312.3	107.7	0.1391	0.5213	0.2667	0.7112	2.2794	0.2667	-819.777
22	10	314.6	105.4	0.1794	0.4295	0.4177	0.7265	2.1431	0.4177	-845.8
22	11	306.1	113.9	0.2067	0.4391	0.4707	0.8091	3.1216	0.4707	-856.026
22	12	311.8	108.2	0.1883	0.5933	0.3173	0.8778	2.0669	0.3173	-871.439
22	13	324.4	95.6	0.124	0.4722	0.2626	0.6098	1.4003	0.2626	-812.919
22	14	308.3	111.7	0.1666	0.4568	0.3646	0.7312	2.5542	0.3646	-838.832
22	15	316	104	0.1996	0.5323	0.3749	0.8459	1.735	0.3749	-872.962
22	16	307.1	112.9	0.2019	0.4534	0.4452	0.8085	2.756	0.4452	-862.188
22	17	316.1	103.9	0.1656	0.6054	0.2736	0.8234	2.0261	0.2736	-847.236
22	18	304.8	115.2	0.1703	0.2876	0.5923	0.6074	3.1348	0.5923	-821.306
22	19	302.6	117.4	0.2758	0.4468	0.6172	0.9708	3.5502	0.6172	-888.159
22	20	316.7	103.3	0.1789	0.5983	0.299	0.8461	1.5351	0.299	-867.398
22	21	321.1	98.9	0.147	0.4887	0.3008	0.6823	1.7989	0.3008	-830.576
23	1	311.8	108.2	0.1622	0.5246	0.3091	0.7665	2.1056	0.3091	-848.992
23	2	316.6	103.4	0.1811	0.4581	0.3954	0.748	1.6487	0.3954	-860.019
23	3	310.2	109.8	0.1852	0.4837	0.3829	0.7896	2.1259	0.3829	-863.591
23	4	304.8	115.2	0.1713	0.4738	0.3616	0.7628	2.3496	0.3616	-855.264
23	5	314.2	105.8	0.1908	0.5695	0.3351	0.8586	1.8703	0.3351	-870.331
23	6	305	115	0.1893	0.4872	0.3884	0.8126	2.6529	0.3884	-862.227
23	7	307.2	112.8	0.1993	0.5212	0.3823	0.8571	2.5189	0.3823	-871.431
23	8	310.8	109.2	0.2083	0.5581	0.3731	0.8976	2.0943	0.3731	-883.027
23	9	308.8	111.2	0.1868	0.5718	0.3268	0.8664	2.3108	0.3268	-866.295
23	10	314.1	105.9	0.193	0.4642	0.4159	0.7842	1.85	0.4159	-866.479
23	11	308.2	111.8	0.1975	0.5036	0.3922	0.8369	2.3496	0.3922	-870.811
23	12	304.1	115.9	0.1846	0.4994	0.3696	0.8145	2.5905	0.3696	-861.624
23	13	317.9	102.1	0.1991	0.5009	0.3975	0.8174	1.5601	0.3975	-872.547
23	14	309.9	110.1	0.2243	0.5124	0.4376	0.8995	1.9967	0.4376	-885.224
23	15	311.7	108.3	0.1835	0.5584	0.3286	0.8403	1.7778	0.3286	-871.514
23	16	307.4	112.6	0.2005	0.4662	0.43	0.8151	2.2679	0.43	-871.806
23	17	315.8	104.2	0.1756	0.5666	0.31	0.8179	1.6951	0.31	-861.267
23	18	301.6	118.4	0.2248	0.364	0.6176	0.7921	3.1361	0.6176	-869.001

23	19	307.7	112.3	0.1832	0.4474	0.4095	0.7614	2.3166	0.4095	-856.712
23	20	309	111	0.1896	0.4881	0.3884	0.8053	1.8437	0.3884	-870.673
23	21	318.7	101.3	0.1613	0.5105	0.316	0.7366	1.6929	0.316	-849.193
23	22	315.6	104.4	0.1918	0.4638	0.4135	0.7783	1.6181	0.4135	-867.63
24	1	315.5	104.5	0.1699	0.529	0.3212	0.7776	1.9543	0.3212	-848.401
24	2	306.9	113.1	0.1598	0.4462	0.3581	0.7108	2.9848	0.3581	-834.615
24	3	303.2	116.8	0.1668	0.4416	0.3777	0.7297	3.3401	0.3777	-838.009
24	4	313.3	106.7	0.1747	0.4833	0.3615	0.7593	1.7003	0.3615	-862.434
24	5	320.1	99.9	0.162	0.6234	0.2598	0.8151	1.5854	0.2598	-853.858
24	6	306.7	113.3	0.1434	0.5002	0.2866	0.7189	2.6351	0.2866	-828.816
24	7	309.8	110.2	0.1421	0.3902	0.364	0.6215	2.4228	0.364	-822.34
24	8	312.2	107.8	0.1879	0.4543	0.4136	0.7689	2.1609	0.4136	-858.025
24	9	303.7	116.3	0.1568	0.6079	0.2579	0.8452	3.2032	0.2579	-838.838
24	10	321.8	98.2	0.1569	0.4624	0.3393	0.6851	1.4217	0.3393	-841.399
24	11	310.1	109.9	0.1899	0.4845	0.3918	0.8008	2.3452	0.3918	-860.529
24	12	304.7	115.3	0.2205	0.4533	0.4864	0.8533	2.7637	0.4864	-876.251
24	13	312.1	107.9	0.1271	0.4131	0.3077	0.6016	2.3012	0.3077	-808.351
24	14	301.6	118.4	0.1976	0.3935	0.5021	0.7584	3.3059	0.5021	-851.573
24	15	309.4	110.6	0.1927	0.5288	0.3645	0.8438	2.2285	0.3645	-868.717
24	16	306.7	113.3	0.1809	0.4282	0.4226	0.7428	2.5909	0.4226	-850.745
24	17	315.4	104.6	0.1341	0.5951	0.2254	0.7468	1.9078	0.2254	-830.535
24	18	300.4	119.6	0.1592	0.3933	0.4047	0.6776	3.6523	0.4047	-827.915
24	19	304.8	115.2	0.2177	0.3745	0.5813	0.7821	2.9682	0.5813	-861.237
24	20	312.5	107.5	0.1621	0.5694	0.2847	0.799	1.7381	0.2847	-858.476
24	21	321.1	98.9	0.1443	0.5968	0.2418	0.7526	1.66	0.2418	-839.866
24	22	310.8	109.2	0.1261	0.4609	0.2735	0.6394	2.2395	0.2735	-815.451
24	23	315.5	104.5	0.161	0.606	0.2657	0.8152	1.5386	0.2657	-860.475
25	1	302.8	117.2	0.1366	0.4356	0.3136	0.6602	3.4332	0.3136	-812.103
25	2	310	110	0.2026	0.4754	0.4261	0.8222	2.572	0.4261	-863.339
25	3	308.9	111.1	0.1657	0.4383	0.3781	0.7134	2.5048	0.3781	-836.482
25	4	304.3	115.7	0.1742	0.4487	0.3883	0.7496	2.7163	0.3883	-849.38
25	5	319.4	100.6	0.1313	0.4955	0.265	0.6556	1.6066	0.265	-822.021
25	6	306.4	113.6	0.1631	0.5839	0.2794	0.8308	2.8035	0.2794	-845.664
25	7	311.5	108.5	0.1365	0.392	0.3482	0.6075	2.2182	0.3482	-816.766
25	8	313.6	106.4	0.1655	0.6024	0.2747	0.8284	2.0252	0.2747	-856.338
25	9	303	117	0.1368	0.6291	0.2174	0.8217	3.4365	0.2174	-823.116
25	10	311.8	108.2	0.1456	0.4534	0.3212	0.6748	2.2614	0.3212	-826.305
25	11	310	110	0.1628	0.493	0.3303	0.748	2.3273	0.3303	-842.648
25	12	304.1	115.9	0.1831	0.5832	0.314	0.8805	2.8736	0.314	-862.293
25	13	315.2	104.8	0.1733	0.4984	0.3477	0.7633	1.9943	0.3477	-846.951
25	14	305.2	114.8	0.2254	0.5138	0.4388	0.9126	2.7761	0.4388	-876.433
25	15	311.1	108.9	0.1829	0.6699	0.2731	0.9275	2.0546	0.2731	-874.493
25	16	306.5	113.5	0.176	0.4238	0.4153	0.7288	2.6459	0.4153	-846.499
25	17	309.2	110.8	0.1526	0.5412	0.2819	0.7651	2.5889	0.2819	-833.66
25	18	297.7	122.3	0.1706	0.413	0.4131	0.7236	4.3226	0.4131	-830.358
25	19	306.2	113.8	0.2178	0.4399	0.4952	0.8339	2.749	0.4952	-869.139
25	20	313.2	106.8	0.1849	0.484	0.3821	0.783	1.6849	0.3821	-863.839
25	21	321.8	98.2	0.1045	0.4829	0.2163	0.5788	1.5552	0.2163	-799.515
25	22	316.5	103.5	0.1506	0.482	0.3124	0.6967	1.7106	0.3124	-833.654
25	23	307.4	112.6	0.1775	0.5218	0.3401	0.8093	2.2858	0.3401	-861.007
25	24	309.1	110.9	0.1711	0.5064	0.3378	0.779	2.3166	0.3378	-851.174
26	1	314.8	105.2	0.1974	0.7115	0.2775	0.9786	1.9656	0.2775	-879.489
26	2	316.6	103.4	0.2178	0.5892	0.3696	0.9276	1.8616	0.3696	-883.696
26	3	312.5	107.5	0.1993	0.567	0.3516	0.8802	2.0915	0.3516	-873.357
26	4	308.8	111.2	0.1941	0.6973	0.2784	0.9821	2.0939	0.2784	-883.072
26	5	327.6	92.4	0.1595	0.9073	0.1758	0.9718	1.0815	0.1758	-869.404
26	6	315.1	104.9	0.1814	0.6541	0.2774	0.8984	1.7612	0.2774	-870.315
26	7	318.1	101.9	0.1848	0.6732	0.2745	0.9099	1.5822	0.2745	-876.119
26	8	320.4	99.6	0.1673	0.7568	0.2211	0.9212	1.4501	0.2211	-867.501
26	9	313.8	106.2	0.1965	0.9207	0.2134	1.1386	2.0038	0.2134	-886.723
26	10	318.6	101.4	0.2081	0.6331	0.3286	0.9319	1.6281	0.3286	-888.427
26	11	315.6	104.4	0.1859	0.7049	0.2636	0.9446	1.7666	0.2636	-879.238
26	12	310.5	109.5	0.2065	0.7708	0.2679	1.0609	2.0592	0.2679	-892.979
26	13	314.7	105.3	0.1896	0.679	0.2792	0.9369	2.0906	0.2792	-872.945

26	14	314.5	105.5	0.1887	0.6457	0.2922	0.9105	1.7673	0.2922	-870.496
26	15	313.2	106.8	0.1782	0.6077	0.2932	0.8621	1.8388	0.2932	-866.622
26	16	311.3	108.7	0.1529	0.734	0.2083	0.9099	2.0808	0.2083	-859.337
26	17	322.8	97.2	0.1655	0.7314	0.2262	0.8894	1.3201	0.2262	-862.699
26	18	306.7	113.3	0.2173	0.5578	0.3896	0.9276	2.6845	0.3896	-883.278
26	19	310.2	109.8	0.2024	0.5705	0.3548	0.8959	2.2497	0.3548	-876.446
26	20	320	100	0.155	0.7456	0.2079	0.8871	1.1487	0.2079	-866.301
26	21	324.5	95.5	0.1731	0.7387	0.2343	0.9051	1.3783	0.2343	-870.776
26	22	316.8	103.2	0.1711	0.6456	0.265	0.863	1.692	0.265	-862.311
26	23	310.6	109.4	0.2061	0.6882	0.2995	0.9951	1.9099	0.2995	-891.671
26	24	311.3	108.7	0.2061	0.6907	0.2983	0.9947	2.0637	0.2983	-889.053
26	25	317.1	102.9	0.1785	0.7546	0.2366	0.9588	1.5359	0.2366	-876.037
27	1	311.1	108.9	0.1415	0.4346	0.3255	0.6523	2.8718	0.3255	-818.208
27	2	318.6	101.4	0.1809	0.5282	0.3426	0.7942	2.1552	0.3426	-853.243
27	3	311.2	108.8	0.2084	0.5028	0.4145	0.854	2.7078	0.4145	-868.066
27	4	311.8	108.2	0.175	0.4807	0.364	0.7611	2.0994	0.364	-855.603
27	5	316.8	103.2	0.1941	0.5995	0.3239	0.8812	2.0568	0.3239	-868.478
27	6	310.4	109.6	0.1612	0.5047	0.3195	0.7525	2.7063	0.3195	-838.838
27	7	313.1	106.9	0.1755	0.4902	0.358	0.7668	2.4722	0.358	-851.912
27	8	313.4	106.6	0.2141	0.5555	0.3854	0.9023	2.3417	0.3854	-876.965
27	9	306.3	113.7	0.1777	0.651	0.273	0.9177	3.3714	0.273	-853.588
27	10	318.2	101.8	0.2055	0.5099	0.4029	0.8376	2.0694	0.4029	-872.215
27	11	314.4	105.6	0.1881	0.5133	0.3665	0.8096	2.4189	0.3665	-858.075
27	12	302.2	117.8	0.2058	0.4056	0.5075	0.7856	3.7023	0.5075	-852.706
27	13	321	99	0.1807	0.5606	0.3223	0.8107	1.892	0.3223	-856.928
27	14	309.6	110.4	0.2253	0.4502	0.5005	0.8533	2.7596	0.5005	-870.449
27	15	309.2	110.8	0.1789	0.6172	0.2898	0.8835	2.6938	0.2898	-858.975
27	16	308.8	111.2	0.1912	0.5033	0.3799	0.8215	2.9509	0.3799	-861.384
27	17	318.5	101.5	0.1862	0.441	0.4221	0.7433	2.0472	0.4221	-848.934
27	18	305.5	114.5	0.217	0.4951	0.4383	0.8785	3.6476	0.4383	-866.972
27	19	306.7	113.3	0.1889	0.3458	0.5463	0.6937	3.3592	0.5463	-835.416
27	20	317.5	102.5	0.2184	0.5979	0.3652	0.9329	1.6432	0.3652	-893.594
27	21	320.5	99.5	0.1667	0.5128	0.3251	0.7461	2.1576	0.3251	-846.71
27	22	313	107	0.1715	0.4871	0.3522	0.7557	2.3955	0.3522	-845.962
27	23	311.5	108.5	0.1642	0.4307	0.3812	0.6992	2.1609	0.3812	-843.898
27	24	314.6	105.4	0.1852	0.4437	0.4173	0.7501	2.0236	0.4173	-857.321
27	25	309.1	110.9	0.154	0.4384	0.3513	0.6874	2.6634	0.3513	-831.287
27	26	309.9	110.1	0.2167	0.5689	0.3809	0.9269	2.6223	0.3809	-875.853
28	1	323	97	0.1811	0.7202	0.2515	0.917	1.5196	0.2515	-866.928
28	2	318.5	101.5	0.2168	0.5008	0.433	0.8565	1.908	0.433	-874.104
28	3	315.8	104.2	0.1851	0.4005	0.462	0.7156	1.9551	0.462	-849.166
28	4	312.5	107.5	0.1898	0.6687	0.2838	0.937	1.9386	0.2838	-876.14
28	5	326.6	93.4	0.1695	0.7917	0.2141	0.9235	1.2734	0.2141	-864.122
28	6	312.9	107.1	0.2006	0.6006	0.3339	0.9078	2.1406	0.3339	-872.52
28	7	316.2	103.8	0.1904	0.5486	0.3471	0.837	1.9549	0.3471	-864.835
28	8	317.6	102.4	0.1877	0.5824	0.3223	0.8519	1.8218	0.3223	-864.494
28	9	316.4	103.6	0.2114	0.7612	0.2777	1.0409	1.9034	0.2777	-885.313
28	10	320.2	99.8	0.2034	0.775	0.2625	1.0176	1.6957	0.2625	-885.178
28	11	322.1	97.9	0.1524	0.6428	0.2371	0.8002	1.4589	0.2371	-850.241
28	12	315.2	104.8	0.1968	0.6396	0.3077	0.9219	1.7936	0.3077	-880.407
28	13	325	95	0.1741	0.6317	0.2756	0.833	1.3818	0.2756	-857.951
28	14	315.3	104.7	0.2067	0.5697	0.3628	0.8916	1.8953	0.3628	-873.918
28	15	319.3	100.7	0.1539	0.5428	0.2836	0.7416	1.5193	0.2836	-844.59
28	16	313.7	106.3	0.1481	0.6681	0.2216	0.8391	2.0728	0.2216	-850.896
28	17	318.4	101.6	0.1785	0.7164	0.2491	0.9258	1.8274	0.2491	-862.743
28	18	311.3	108.7	0.1944	0.451	0.431	0.7824	2.3662	0.431	-858.005
28	19	311.4	108.6	0.2398	0.5802	0.4133	0.9836	2.3597	0.4133	-892.535
28	20	319.9	100.1	0.1736	0.7586	0.2288	0.9388	1.3461	0.2288	-874.592
28	21	326.5	93.5	0.1493	0.595	0.2509	0.7454	1.4136	0.2509	-840.024
28	22	324	96	0.1582	0.4667	0.339	0.6862	1.3321	0.339	-837.873
28	23	316.1	103.9	0.2038	0.6361	0.3204	0.932	1.6293	0.3204	-885.964
28	24	312.4	107.6	0.1666	0.624	0.267	0.8514	2.1648	0.267	-855.553
28	25	317.2	102.8	0.1559	0.7673	0.2031	0.9168	1.698	0.2031	-855.869
28	26	316.2	103.8	0.1443	0.6914	0.2086	0.8386	1.7728	0.2086	-844.141

28	27	319.1	100.9	0.1877	0.6027	0.3114	0.8623	1.8315	0.3114	-865.543
29	1	300.6	119.4	0.1483	0.5636	0.2631	0.7988	4.038	0.2631	-827.644
29	2	309.3	110.7	0.1972	0.5024	0.3925	0.833	2.7035	0.3925	-864.67
29	3	304.7	115.3	0.1861	0.5186	0.3589	0.8321	3.0208	0.3589	-861.246
29	4	303.3	116.7	0.1648	0.6583	0.2504	0.906	2.7309	0.2504	-862.835
29	5	313.4	106.6	0.1514	0.5602	0.2702	0.7655	2.0579	0.2702	-844.005
29	6	299.7	120.3	0.1571	0.4665	0.3367	0.737	3.7232	0.3367	-831.718
29	7	303	117	0.1581	0.5531	0.2858	0.8043	3.3932	0.2858	-842.816
29	8	310.4	109.6	0.1724	0.5894	0.2925	0.8436	2.2684	0.2925	-861.453
29	9	301.8	118.2	0.1823	0.5982	0.3047	0.8981	3.4565	0.3047	-857.782
29	10	311.7	108.3	0.1581	0.51	0.3101	0.7466	2.2725	0.3101	-844.599
29	11	298.6	121.4	0.1922	0.566	0.3395	0.9006	4.5049	0.3395	-856.24
29	12	295.3	124.7	0.186	0.5967	0.3117	0.9236	4.4105	0.3117	-858.877
29	13	313.9	106.1	0.1796	0.6185	0.2903	0.8714	2.0958	0.2903	-864.685
29	14	303.2	116.8	0.224	0.5479	0.4088	0.9423	3.0065	0.4088	-881.744
29	15	303.4	116.6	0.157	0.6841	0.2295	0.9099	2.8896	0.2295	-856.207
29	16	305.6	114.4	0.1465	0.4753	0.3083	0.7082	2.7087	0.3083	-834.772
29	17	308.9	111.1	0.1548	0.6709	0.2307	0.8737	2.5661	0.2307	-850.959
29	18	301	119	0.185	0.4311	0.4291	0.7642	3.6536	0.4291	-851.013
29	19	300.5	119.5	0.2017	0.4725	0.427	0.8362	3.7334	0.427	-859.779
29	20	304.2	115.8	0.2039	0.7176	0.2841	1.0366	2.58	0.2841	-889.549
29	21	309.9	110.1	0.1395	0.6409	0.2177	0.8128	2.9128	0.2177	-835.857
29	22	305.4	114.6	0.1797	0.5692	0.3158	0.8579	2.8118	0.3158	-860.076
29	23	300.7	119.3	0.2	0.5219	0.3833	0.8744	3.1465	0.3833	-871.566
29	24	307.6	112.4	0.1628	0.4223	0.3854	0.6968	2.4289	0.3854	-840.401
29	25	306.7	113.3	0.1459	0.5057	0.2886	0.7289	2.4765	0.2886	-838.579
29	26	304.3	115.7	0.2085	0.6664	0.3129	1.0039	2.8114	0.3129	-884.957
29	27	305.8	114.2	0.1632	0.5966	0.2736	0.8432	3.2156	0.2736	-848.35
29	28	309.6	110.4	0.1976	0.694	0.2848	0.9845	2.43	0.2848	-881.448
30	1	309.4	110.6	0.2021	0.5738	0.3522	0.8998	2.719	0.3522	-867.678
30	2	313.9	106.1	0.2235	0.4353	0.5135	0.831	2.3014	0.5135	-871.639
30	3	309.7	110.3	0.1804	0.4912	0.3672	0.786	2.5772	0.3672	-854.22
30	4	306	114	0.2055	0.5215	0.3941	0.8738	2.5765	0.3941	-875.663
30	5	320.9	99.1	0.1805	0.6033	0.2991	0.8407	1.5765	0.2991	-864.033
30	6	308.4	111.6	0.1982	0.494	0.4013	0.8304	2.6441	0.4013	-862.87
30	7	311.2	108.8	0.1881	0.405	0.4645	0.733	2.3816	0.4645	-852.082
30	8	315.1	104.9	0.1666	0.6149	0.271	0.8359	1.9518	0.271	-855.84
30	9	309.8	110.2	0.1996	0.6996	0.2853	0.9922	2.5143	0.2853	-875.791
30	10	311.6	108.4	0.1886	0.452	0.4173	0.7698	2.4759	0.4173	-854.108
30	11	315.3	104.7	0.1879	0.5989	0.3137	0.8711	1.9737	0.3137	-873.473
30	12	304.2	115.8	0.212	0.5458	0.3884	0.9122	2.9859	0.3884	-875.655
30	13	316.8	103.2	0.2023	0.511	0.3959	0.8347	2.0084	0.3959	-868.066
30	14	306.5	113.5	0.2022	0.4689	0.4312	0.8228	2.8	0.4312	-859.093
30	15	306.4	113.6	0.1668	0.5486	0.3041	0.8103	2.757	0.3041	-850.25
30	16	306.4	113.6	0.1859	0.5579	0.3333	0.8596	2.8263	0.3333	-863.993
30	17	321.2	98.8	0.149	0.6076	0.2452	0.7705	1.5739	0.2452	-841.97
30	18	302.7	117.3	0.2355	0.3687	0.6388	0.8182	3.4941	0.6388	-868.114
30	19	308.4	111.6	0.224	0.4675	0.4791	0.8661	2.6309	0.4791	-876.524
30	20	314.5	105.5	0.204	0.4693	0.4348	0.8119	1.6948	0.4348	-875.006
30	21	320.3	99.7	0.1804	0.5438	0.3318	0.8001	1.8754	0.3318	-860.854
30	22	307.2	112.8	0.1991	0.3956	0.5032	0.7555	2.8322	0.5032	-849.064
30	23	311.9	108.1	0.2079	0.4365	0.4762	0.8002	1.8923	0.4762	-872.968
30	24	306.6	113.4	0.181	0.4923	0.3676	0.7951	2.7051	0.3676	-854.173
30	25	310.2	109.8	0.194	0.5023	0.3862	0.8237	2.3104	0.3862	-865.251
30	26	313.7	106.3	0.157	0.5323	0.2948	0.7559	1.9367	0.2948	-848.359
30	27	309	111	0.2133	0.4711	0.4527	0.8441	2.9427	0.4527	-864.854
30	28	309.5	110.5	0.1485	0.5348	0.2777	0.7503	2.6044	0.2777	-832.195
30	29	303.9	116.1	0.1794	0.6218	0.2885	0.9051	3.0724	0.2885	-862.743
31	1	312.5	107.5	0.2459	0.4544	0.5411	0.8977	2.2589	0.5411	-882.256
31	2	313.2	106.8	0.2218	0.4452	0.4982	0.8357	2.3308	0.4982	-871.29
31	3	307.3	112.7	0.2192	0.4772	0.4594	0.8655	2.7496	0.4594	-873.719
31	4	306.2	113.8	0.24	0.5852	0.4101	1.0006	2.3596	0.4101	-900.475
31	5	317.7	102.3	0.2325	0.6952	0.3345	1.0356	1.7284	0.3345	-897.238
31	6	310.9	109.1	0.2107	0.4996	0.4217	0.8572	2.1482	0.4217	-874.454

31	7	308.8	111.2	0.2229	0.4424	0.5039	0.843	2.5715	0.5039	-873.046
31	8	315.9	104.1	0.2422	0.528	0.4588	0.9392	1.8002	0.4588	-893.992
31	9	308.8	111.2	0.26	0.5383	0.4829	1.001	2.5015	0.4829	-897.821
31	10	312.6	107.4	0.2403	0.5129	0.4684	0.93	2.2598	0.4684	-888.014
31	11	310	110	0.2557	0.5424	0.4714	0.9924	2.4568	0.4714	-897.992
31	12	310.4	109.6	0.249	0.4555	0.5466	0.9085	2.021	0.5466	-894.379
31	13	314.6	105.4	0.21	0.5043	0.4164	0.8515	2.1602	0.4164	-872.985
31	14	318.9	101.1	0.0156	0.0556	0.2805	0.0757	1.4301	0.2805	-623.707
31	15	310.8	109.2	0.2109	0.6604	0.3193	0.9831	2.0473	0.3193	-887.266
31	16	306.2	113.8	0.2367	0.4629	0.5115	0.894	2.7211	0.5115	-884.503
31	17	315	105	0.2226	0.5068	0.4393	0.881	2.008	0.4393	-876.214
31	18	299.9	120.1	0.2568	0.3928	0.6538	0.8871	3.9941	0.6538	-875.939
31	19	308.2	111.8	0.32	0.4198	0.7622	1.0396	2.5236	0.7622	-915.394
31	20	312.8	107.2	0.2118	0.4093	0.5175	0.7866	1.7096	0.5175	-871.837
31	21	323.4	96.6	0.2272	0.5719	0.3972	0.9194	1.547	0.3972	-889.327
31	22	310.9	109.1	0.1737	0.4421	0.3929	0.7303	2.2393	0.3929	-845.351
31	23	312.7	107.3	0.2338	0.5253	0.445	0.9247	1.7019	0.445	-895.206
31	24	302.5	117.5	0.2078	0.4548	0.4569	0.8307	3.1531	0.4569	-866.192
31	25	306.8	113.2	0.2342	0.5059	0.4629	0.9222	2.5308	0.4629	-884.535
31	26	314.7	105.3	0.1981	0.7046	0.2811	0.975	1.7092	0.2811	-882.191
31	27	312.8	107.2	0.2337	0.4842	0.4826	0.8929	2.3419	0.4826	-882.742
31	28	317.7	102.3	0.2114	0.5621	0.3761	0.8905	1.6847	0.3761	-879.088
31	29	305.7	114.3	0.2386	0.5764	0.4138	0.9916	2.6435	0.4138	-895.371
31	30	308.1	111.9	0.2117	0.4623	0.4579	0.8352	2.5496	0.4579	-866.838
32	1	319.7	100.3	0.1815	0.5745	0.3159	0.826	1.912	0.3159	-858.329
32	2	318.5	101.5	0.1892	0.458	0.4132	0.7625	2.2491	0.4132	-850.556
32	3	313.7	106.3	0.2155	0.4645	0.4641	0.8356	2.3929	0.4641	-869.686
32	4	312.5	107.5	0.213	0.6342	0.3358	0.9624	2.0512	0.3358	-888.939
32	5	318.2	101.8	0.2046	0.6139	0.3332	0.9114	1.9445	0.3332	-874.638
32	6	313.8	106.2	0.1907	0.5023	0.3797	0.8086	2.3019	0.3797	-855.566
32	7	315.7	104.3	0.1897	0.4217	0.4498	0.7419	2.2064	0.4498	-854.327
32	8	317.6	102.4	0.2026	0.6002	0.3376	0.8988	1.9554	0.3376	-876.499
32	9	312.2	107.8	0.19	0.5025	0.3781	0.8105	2.5249	0.3781	-856.262
32	10	317	103	0.2015	0.4528	0.4451	0.7894	2.2318	0.4451	-859.229
32	11	317.4	102.6	0.1973	0.4632	0.426	0.7869	2.0977	0.426	-858.622
32	12	312.2	107.8	0.1958	0.6734	0.2908	0.9554	2.198	0.2908	-877.089
32	13	326	94	0.1722	0.4721	0.3648	0.7181	1.4959	0.3648	-842.593
32	14	312.2	107.8	0.2158	0.425	0.5077	0.8084	2.4355	0.5077	-862.396
32	15	317.7	102.3	0.1878	0.5844	0.3214	0.8531	1.7607	0.3214	-867.211
32	16	308.7	111.3	0.2065	0.3417	0.6042	0.7269	3.0432	0.6042	-850.881
32	17	324.1	95.9	0.1769	0.5222	0.3388	0.7674	1.5399	0.3388	-850.594
32	18	310.6	109.4	0.2571	0.3195	0.8045	0.82	2.8522	0.8045	-876.071
32	19	309.8	110.2	0.2389	0.4603	0.5191	0.8911	2.8739	0.5191	-877.963
32	20	319.3	100.7	0.1836	0.5318	0.3452	0.8013	1.5258	0.3452	-864.127
32	21	325.5	94.5	0.2037	0.4431	0.4598	0.7728	1.6947	0.4598	-861.519
32	22	315.1	104.9	0.1857	0.4433	0.419	0.7502	2.1936	0.419	-851.929
32	23	314.3	105.7	0.1968	0.5352	0.3676	0.8459	1.9082	0.3676	-872.624
32	24	310.4	109.6	0.1943	0.4265	0.4556	0.7646	2.5327	0.4556	-852.095
32	25	316.1	103.9	0.2051	0.5014	0.4089	0.8352	1.8766	0.4089	-870.153
32	26	317.9	102.1	0.2328	0.5577	0.4175	0.9355	1.7543	0.4175	-890.951
32	27	318.8	101.2	0.213	0.5563	0.3829	0.8871	2.1397	0.3829	-877.268
32	28	326.9	93.1	0.1667	0.7165	0.2326	0.8656	1.2593	0.2326	-861.14
32	29	308.4	111.6	0.2205	0.4441	0.4965	0.8397	2.817	0.4965	-871.308
32	30	315.9	104.1	0.1843	0.4915	0.375	0.7814	2.1437	0.375	-857.647
32	31	316.3	103.7	0.2216	0.4304	0.5149	0.8195	1.9866	0.5149	-871.067
33	1	311	109	0.1722	0.4968	0.3467	0.7693	2.3466	0.3467	-850.183
33	2	309.2	110.8	0.1764	0.5088	0.3468	0.7924	2.7349	0.3468	-851.328
33	3	308.4	111.6	0.1899	0.5013	0.3789	0.8181	2.5636	0.3789	-864.422
33	4	302.4	117.6	0.1673	0.6956	0.2405	0.9455	2.9491	0.2405	-863.398
33	5	312.1	107.9	0.1781	0.7682	0.2318	0.9891	2.1942	0.2318	-871.789
33	6	309.6	110.4	0.1398	0.5546	0.252	0.7465	2.3115	0.252	-831.743
33	7	305.7	114.3	0.149	0.5795	0.2571	0.7985	2.984	0.2571	-842.445
33	8	312.8	107.2	0.1803	0.5125	0.3518	0.7953	2.0635	0.3518	-861.391
33	9	306.8	113.2	0.1735	0.6424	0.2701	0.8997	2.8102	0.2701	-858.73

33	10	310.9	109.1	0.145	0.5169	0.2806	0.7248	2.3795	0.2806	-832.539
33	11	309.3	110.7	0.1784	0.5258	0.3392	0.8098	2.4348	0.3392	-857.188
33	12	302.3	117.7	0.1962	0.4521	0.4339	0.8037	3.1236	0.4339	-860.303
33	13	316.4	103.6	0.1457	0.5584	0.261	0.7425	1.8646	0.261	-835.842
33	14	307.1	112.9	0.2474	0.47	0.5263	0.9216	2.5265	0.5263	-889.34
33	15	309.6	110.4	0.1785	0.5364	0.3328	0.818	2.1803	0.3328	-863.193
33	16	307.6	112.4	0.1826	0.4263	0.4284	0.7435	2.4936	0.4284	-852.918
33	17	312.5	107.5	0.1319	0.5764	0.2288	0.7369	2.2073	0.2288	-829.376
33	18	299.6	120.4	0.1507	0.4595	0.328	0.7177	3.9692	0.328	-829.106
33	19	305.5	114.5	0.212	0.5248	0.4039	0.8917	2.864	0.4039	-874.171
33	20	310.4	109.6	0.1675	0.6478	0.2586	0.8786	1.9111	0.2586	-864.431
33	21	310.5	109.5	0.145	0.5181	0.2798	0.7269	2.8054	0.2798	-829.993
33	22	313.2	106.8	0.1603	0.4459	0.3595	0.6989	1.9756	0.3595	-841.289
33	23	306.7	113.3	0.1849	0.5043	0.3666	0.8132	2.3208	0.3666	-866.741
33	24	309.9	110.1	0.1289	0.5777	0.2231	0.7397	2.1802	0.2231	-830.077
33	25	303	117	0.146	0.5754	0.2538	0.7968	3.1284	0.2538	-836.13
33	26	310.3	109.7	0.199	0.7122	0.2794	0.999	2.1238	0.2794	-886.7
33	27	308.4	111.6	0.1968	0.5228	0.3765	0.8504	2.8123	0.3765	-865.192
33	28	313.5	106.5	0.1893	0.6113	0.3096	0.889	1.9976	0.3096	-870.222
33	29	304.3	115.7	0.154	0.5633	0.2733	0.8002	2.8027	0.2733	-848.368
33	30	307.7	112.3	0.1842	0.5933	0.3104	0.8808	2.5999	0.3104	-868.406
33	31	308.5	111.5	0.2574	0.4624	0.5567	0.9355	2.3179	0.5567	-896.932
33	32	312.2	107.8	0.1995	0.5626	0.3545	0.878	2.3119	0.3545	-873.093
34	1	308.8	111.2	0.2022	0.6396	0.3161	0.9538	2.6834	0.3161	-873.248
34	2	314.1	105.9	0.2263	0.5018	0.451	0.8873	2.1044	0.451	-886.447
34	3	307.1	112.9	0.2181	0.539	0.4046	0.9131	2.6666	0.4046	-881.783
34	4	305	115	0.2208	0.6732	0.3279	1.034	2.5203	0.3279	-896.055
34	5	312	108	0.2269	0.6407	0.3542	1.0001	2.2083	0.3542	-891.415
34	6	307.8	112.2	0.1911	0.6314	0.3026	0.9262	2.3474	0.3026	-874.065
34	7	314.1	105.9	0.2	0.4985	0.4011	0.8257	1.9144	0.4011	-874.091
34	8	311.4	108.6	0.2059	0.5638	0.3652	0.8954	2.1886	0.3652	-879.916
34	9	309.7	110.3	0.2101	0.6905	0.3043	1.0087	2.3112	0.3043	-885.834
34	10	313.5	106.5	0.2024	0.5704	0.3549	0.8872	2.0884	0.3549	-875.431
34	11	306.6	113.4	0.217	0.6305	0.3442	0.986	2.8406	0.3442	-884.07
34	12	308.5	111.5	0.232	0.6502	0.3568	1.0291	2.1618	0.3568	-902.502
34	13	316.2	103.8	0.1771	0.6113	0.2897	0.8532	1.8597	0.2897	-862.537
34	14	306.8	113.2	0.2528	0.5086	0.4971	0.9652	2.501	0.4971	-896.205
34	15	304.9	115.1	0.1829	0.5967	0.3066	0.8888	2.6785	0.3066	-870.147
34	16	307.1	112.9	0.21	0.5655	0.3714	0.9166	2.534	0.3714	-881.484
34	17	309.7	110.3	0.1826	0.9613	0.1899	1.161	2.4433	0.1899	-880.18
34	18	301.9	118.1	0.213	0.5029	0.4234	0.8835	3.5192	0.4234	-870.378
34	19	310.5	109.5	0.2662	0.5674	0.4692	1.0342	2.1858	0.4692	-910.854
34	20	305.4	114.6	0.2055	0.7044	0.2917	1.025	2.4329	0.2917	-889.305
34	21	316.5	103.5	0.1642	0.7179	0.2287	0.902	2.0705	0.2287	-860.602
34	22	311.5	108.5	0.2019	0.5774	0.3497	0.8967	2.0857	0.3497	-878.285
34	23	306	114	0.2079	0.5392	0.3856	0.8935	2.3877	0.3856	-882.749
34	24	306.7	113.3	0.207	0.5456	0.3795	0.895	2.5725	0.3795	-877.882
34	25	306.5	113.5	0.1915	0.5641	0.3394	0.8765	2.5095	0.3394	-870.269
34	26	309.2	110.8	0.2199	0.6711	0.3277	1.0169	2.178	0.3277	-896.81
34	27	313.3	106.7	0.2518	0.6935	0.363	1.0919	2.1604	0.363	-912.103
34	28	310.4	109.6	0.2096	0.6057	0.3461	0.9391	2.3511	0.3461	-880.612
34	29	305.9	114.1	0.1953	0.5157	0.3787	0.8469	2.6267	0.3787	-869.072
34	30	307.1	112.9	0.1997	0.5768	0.3463	0.9031	2.628	0.3463	-875.441
34	31	308	112	0.2615	0.5472	0.478	1.0132	2.3681	0.478	-905.488
34	32	312.8	107.2	0.2456	0.6071	0.4046	1.0137	2.232	0.4046	-897.441
34	33	310.5	109.5	0.17	0.6285	0.2705	0.8685	2.0102	0.2705	-866.453
35	1	310.2	109.8	0.194	0.5346	0.3629	0.8491	2.8156	0.3629	-857.047
35	2	313.2	106.8	0.2049	0.5245	0.3906	0.8584	2.6982	0.3906	-863.775
35	3	304.6	115.4	0.1638	0.3989	0.4107	0.6854	3.8058	0.4107	-824.308
35	4	306.9	113.1	0.1909	0.5326	0.3585	0.8488	2.6512	0.3585	-864.958
35	5	316.1	103.9	0.1821	0.6432	0.2831	0.8886	2.0537	0.2831	-861.632
35	6	311.7	108.3	0.1623	0.4682	0.3467	0.7237	2.512	0.3467	-838.467
35	7	310.6	109.4	0.1632	0.4819	0.3387	0.7385	2.6847	0.3387	-839.4
35	8	313.3	106.7	0.1635	0.658	0.2485	0.8675	2.2826	0.2485	-851.6

35	9	307.6	112.4	0.1954	0.4808	0.4063	0.8153	3.1236	0.4063	-853.858
35	10	317.6	102.4	0.1696	0.4901	0.346	0.7432	2.0038	0.346	-845.455
35	11	309.7	110.3	0.1847	0.451	0.4095	0.7639	2.9216	0.4095	-848.198
35	12	306.5	113.5	0.2122	0.5046	0.4206	0.8737	2.8982	0.4206	-871.707
35	13	315.4	104.6	0.1467	0.5706	0.2571	0.7567	2.406	0.2571	-830.628
35	14	306.9	113.1	0.2377	0.5611	0.4237	0.9744	3.0589	0.4237	-884.026
35	15	311.7	108.3	0.1965	0.7046	0.2788	0.9824	2.2896	0.2788	-879.323
35	16	310.5	109.5	0.1336	0.49	0.2727	0.6798	2.5599	0.2727	-823.801
35	17	313.6	106.4	0.1649	0.6709	0.2459	0.8792	2.5277	0.2459	-848.149
35	18	302.6	117.4	0.2057	0.3823	0.5379	0.7651	4.092	0.5379	-847.456
35	19	307.7	112.3	0.2212	0.5233	0.4226	0.9058	3.0072	0.4226	-872.558
35	20	313.1	106.9	0.2029	0.6315	0.3213	0.9361	2.0014	0.3213	-882.123
35	21	322.3	97.7	0.1504	0.5212	0.2886	0.71	1.8123	0.2886	-833.8
35	22	312.4	107.6	0.1692	0.4522	0.3742	0.7252	2.4054	0.3742	-840.179
35	23	307.7	112.3	0.2284	0.5044	0.4529	0.9067	2.5481	0.4529	-885.194
35	24	307.1	112.9	0.1693	0.5087	0.3329	0.7817	2.8513	0.3329	-846.691
35	25	309.6	110.4	0.1666	0.5699	0.2924	0.818	2.5485	0.2924	-846.663
35	26	311.5	108.5	0.1765	0.7785	0.2268	0.996	2.3435	0.2268	-869.353
35	27	311.4	108.6	0.1981	0.5672	0.3493	0.8807	2.9151	0.3493	-862.278
35	28	311.9	108.1	0.148	0.6223	0.2378	0.8101	2.4855	0.2378	-837.243
35	29	306	114	0.1582	0.5997	0.2638	0.8342	2.9884	0.2638	-847.316
35	30	310.9	109.1	0.1659	0.4963	0.3344	0.7553	2.5769	0.3344	-843.257
35	31	306.8	113.2	0.2304	0.549	0.4196	0.9488	3.045	0.4196	-881.136
35	32	315.2	104.8	0.2456	0.5031	0.4882	0.9298	2.4178	0.4882	-888.422
35	33	309.6	110.4	0.1744	0.5096	0.3422	0.7874	2.5388	0.3422	-849.962
35	34	309	111	0.2031	0.6194	0.3279	0.9394	2.5339	0.3279	-879.287
36	1	314.6	105.4	0.1686	0.6512	0.2589	0.8692	2.1017	0.2589	-858.439
36	2	315.3	104.7	0.1989	0.4628	0.4299	0.7941	2.0811	0.4299	-862.517
36	3	311.6	108.4	0.2038	0.6102	0.334	0.926	2.3246	0.334	-876.401
36	4	311.7	108.3	0.1878	0.6565	0.2861	0.926	1.9682	0.2861	-877.907
36	5	319.3	100.7	0.1746	0.7155	0.244	0.9129	1.7178	0.244	-863.415
36	6	311.9	108.1	0.1712	0.5972	0.2867	0.8424	2.2062	0.2867	-855.126
36	7	309.9	110.1	0.168	0.469	0.3582	0.7407	2.5328	0.3582	-844.23
36	8	287.7	132.3	0.0108	0.0235	0.4583	0.0443	8.1142	0.4583	-602.05
36	9	310.6	109.4	0.1793	0.656	0.2734	0.9103	2.4406	0.2734	-857.951

36	10	319.8	100.2	0.1657	0.5271	0.3143	0.7558	1.643	0.3143	-851.984
36	11	312.3	107.7	0.192	0.5361	0.3582	0.8409	2.2369	0.3582	-866.175
36	12	310.5	109.5	0.196	0.4962	0.395	0.8227	2.2161	0.395	-865.682
36	13	322.3	97.7	0.1832	0.5521	0.3319	0.807	1.4968	0.3319	-862.311
36	14	313.5	106.5	0.2408	0.531	0.4535	0.9432	1.9811	0.4535	-891.275
36	15	313.1	106.9	0.2101	0.5011	0.4193	0.8525	1.9973	0.4193	-875.046
36	16	313.7	106.3	0.1737	0.5768	0.3012	0.8272	1.9619	0.3012	-863.616
36	17	316.7	103.3	0.1529	0.7237	0.2113	0.8801	1.9224	0.2113	-849.892
36	18	309.7	110.3	0.1997	0.4819	0.4143	0.8213	2.3907	0.4143	-866.066
36	19	308.1	111.9	0.2338	0.4675	0.5001	0.8883	2.6419	0.5001	-879.887
36	20	316.3	103.7	0.2011	0.6962	0.2889	0.9701	1.5605	0.2889	-890.841
36	21	318	102	0.1584	0.6563	0.2413	0.838	2.0243	0.2413	-851.913
36	22	308.7	111.3	0.189	0.4631	0.4081	0.785	2.6167	0.4081	-850.734
36	23	309.6	110.4	0.2141	0.6106	0.3507	0.9552	2.1846	0.3507	-889.791
36	24	311.2	108.8	0.1924	0.5081	0.3786	0.8226	2.2252	0.3786	-865.42
36	25	313.3	106.7	0.1694	0.6223	0.2722	0.8534	2.0222	0.2722	-859.839
36	26	319.7	100.3	0.1711	0.7773	0.2201	0.9475	1.4615	0.2201	-870.658
36	27	313.5	106.5	0.2128	0.5784	0.3679	0.9163	2.2609	0.3679	-879.201
36	28	317.2	102.8	0.1914	0.6023	0.3178	0.8761	1.8146	0.3178	-868.42
36	29	310	110	0.1762	0.609	0.2893	0.8687	2.2609	0.2893	-865.628
36	30	312.9	107.1	0.1757	0.67	0.2622	0.9052	2.1123	0.2622	-864.715
36	31	313.2	106.8	0.2553	0.5873	0.4347	1.0191	1.981	0.4347	-904.812
36	32	317.7	102.3	0.1952	0.6805	0.2869	0.9403	1.8865	0.2869	-878.767
36	33	311.8	108.2	0.1829	0.5735	0.319	0.8506	2.1191	0.319	-867.664
36	34	310.1	109.9	0.2109	0.6185	0.3409	0.9526	2.2643	0.3409	-886.438
36	35	312.1	107.9	0.1687	0.7165	0.2354	0.9284	2.3727	0.2354	-858.317

Sequence ID Key

1	Cres90_S1
2	Ccan02_S3
3	Ceug56_S4
4	Ctsir35_S6
5	Ctsir36_S7
6	Cmil68_S8
7	Ctsir39_S9
8	Cmil62_S10
9	Cper47_S11
10	Cper52_S12
11	Cbon82_S13
12	Cper54_S15
13	Cper59_S16
14	Cmil64_S17
15	Cmont76_S18
16	Cmil61_S19
17	Candr70_S21
18	Cmont78_S24
19	Cres94_S25
20	Cper60_S26
21	Cbon82_S27
22	Cres92_S28
23	Cres94_S29
24	Cmil62_S32
25	Cmil65_S33
26	Cmont77_S34
27	Candr71_S36
28	Candr72_S37
29	Cpseud121_S38
30	Ctric84_S39
31	Ccan118_S40
32	Ccan118_S41
33	Ccan119_S42
34	Cbon80_S45
35	Ctsir36_S49
36	Cres91_S53
